# Dynamic changes in metabolites of the kynurenine pathway in Alzheimer’s disease, Parkinson’s disease, and Huntington’s disease: A systematic Review and meta-analysis

**DOI:** 10.3389/fimmu.2022.997240

**Published:** 2022-10-03

**Authors:** Mobina Fathi, Kimia Vakili, Shirin Yaghoobpoor, Arian Tavasol, Kimia Jazi, Ramtin Hajibeygi, Sina Shool, Fatemeh Sodeifian, Andis Klegeris, Alyssa McElhinney, Mostafa Rezaei Tavirani, Fatemeh Sayehmiri

**Affiliations:** ^1^ Student Research Committee, Faculty of Medicine, Shahid Beheshti University of Medical Sciences, Tehran, Iran; ^2^ Student Research Committee, Faculty of Medicine, Medical University of Qom, Qom, Iran; ^3^ Department of Neurology, Faculty of Medicine, Tehran Medical Sciences, Islamic Azad University, Tehran, Iran; ^4^ Faculty of Medicine, Shahid Beheshti University of Medical Sciences, Tehran, Iran; ^5^ Department of Biology, Faculty of Science, University of British Columbia Okanagan Campus, Kelowna, BC, Canada; ^6^ Proteomics Research Center, Faculty of Paramedical Sciences, Shahid Beheshti University of Medical Sciences, Tehran, Iran

**Keywords:** alzheimer’s disease, parkinson’s disease, huntington’s disease, kynurenine (KYN), tryptophan, kynurenic acid, anthranilic acid

## Abstract

**Background:**

Tryptophan (TRP) is an essential amino acid that must be provided in the diet. The kynurenine pathway (KP) is the main route of TRP catabolism into nicotinamide adenosine dinucleotide (NAD^+^), and metabolites of this pathway may have protective or degenerative effects on the nervous system. Thus, the KP may be involved in neurodegenerative diseases.

**Objectives:**

The purpose of this systematic review and meta-analysis is to assess the changes in KP metabolites such as TRP, kynurenine (KYN), kynurenic acid (KYNA), Anthranilic acid (AA), 3-hydroxykynurenine (3-HK), 5-Hydroxyindoleacetic acid (5-HIAA), and 3-Hydroxyanthranilic acid (3-HANA) in Alzheimer’s disease (AD), Parkinson’s disease (PD), and Huntington’s disease (HD) patients compared to the control group.

**Methods:**

We conducted a literature search using PubMed/Medline, Scopus, Google Scholar, Web of Science, and EMBASE electronic databases to find articles published up to 2022. Studies measuring TRP, KYN, KYNA, AA, 3-HK, 5-HIAA, 3-HANA in AD, PD, or HD patients and controls were identified. Standardized mean differences (SMDs) were used to determine the differences in the levels of the KP metabolites between the two groups.

**Results:**

A total of 30 studies compromising 689 patients and 774 controls were included in our meta-analysis. Our results showed that the blood levels of TRP was significantly lower in the AD (SMD=-0.68, 95% CI=-0.97 to -0.40, p=0.000, I2 = 41.8%, k=8, n=382), PD (SMD=-0.77, 95% CI=-1.24 to -0.30, p=0.001, I2 = 74.9%, k=4, n=352), and HD (SMD=-0.90, 95% CI=-1.71 to -0.10, p=0.028, I2 = 91.0%, k=5, n=369) patients compared to the controls. Moreover, the CSF levels of 3-HK in AD patients (p=0.020) and the blood levels of KYN in HD patients (p=0.020) were lower compared with controls.

**Conclusion:**

Overall, the findings of this meta-analysis support the hypothesis that the alterations in the KP may be involved in the pathogenesis of AD, PD, and HD. However, additional research is needed to show whether other KP metabolites also vary in AD, PD, and HD patients. So, the metabolites of KP can be used for better diagnosing these diseases.

## Introduction

Tryptophan (TRP) is an essential amino acid. Thus, it must be obtained from the diet (1). TRP is metabolized through different pathways and generates several biologically active products, such as serotonin, melatonin, tryptamine, and nicotinamide (vitamin B_6_) (2, 3). The kynurenine pathway (KP) is the main route involved in TRP catabolism, which produces nicotinamide adenosine dinucleotide (NAD^+^) (4, 5).

Converting TRP to kynurenine (KYN), either through indoleamine 2,3-dioxygenase (IDO) or tryptophan 2,3-dioxygenase (TDO), is the first step in KP (5). The subsequent metabolites produced from this pathway include kynurenic acid (KYNA), 3-hydroxykynurenine (3-HK), and anthranilic acid (AA), which are generated by kynurenine aminotransferase (KATs), kynurenine 3-monooxygenase (KMO), and kynureninase, respectively (6). Cellular expression of the KP is completely present in monocytic lineage cells such as macrophages and microglia (7), but is partially present in astrocytes (8), neurons (9) and endothelial cells (10). Of note, the production of KYNA occurs in astrocytes; however, 3-HK, QUIN, and AA are produced in microglia (11). Furthermore, a study conducted by Sathyasaikumar et al, found KMO only in microglia and neurons but not in astrocytes using isolated mouse cells ex vivo (12). Then, 3-HK and AA are degraded to 3-hydroxyanthranilic acid (3-HANA). Kynureninase catalyzes the conversion of 3-HK to 3-HANA, and subsequently, 3-HANA is oxidized by 3-Hydroxyanthranilic acid oxygenase into quinolinic acid (QUIN), which is ultimately converted to NAD^+^ (4, 5). NAD^+^ is a cofactor for poly (ADP-ribose) polymerase (PARP), which repairs DNA damage (6). Increased activation of PARP due to high DNA damage can be caused by formation of amyloid beta which induces oxidative stress. Overactivation of PARP can also leads to depletion of NAD^+^ and ATP, and thereby cell necrosis resulting in cognitive impairment (13).

Different metabolites produced through KP can affect the neural system in different ways (4). For instance, if the KP shifted toward KYNA production, neuron injuries would be reduced (14). The neuroprotective effects of KYNA are due to the antagonizing effect of KYNA on N-Methyl-D-aspartate (NMDA) receptors, which are ionotropic glutamate receptors, and α7 nicotinic acetylcholine receptors (α7nAChR). KYNA is a competitive inhibitor of the former receptor while inhibiting the latter non-competitively. Moreover, KYNA acts as an antioxidant and a free radical scavenger (15). On the other hand, the formation of some metabolites such as 3-HK, 3-HANA, and QUIN leads to excitotoxic neuronal damage (4). Also, it has been stated in some studies that KP metabolites have positive and negative impacts on several neurodegenerative diseases, including Alzheimer’s disease (AD), Parkinson’s disease (PD), and Huntington’s disease (HD) (16).

AD is the most common human neurodegenerative disorder (17), yet its etiopathogenesis is not understood, as there is more than a single factor responsible for its onset (4, 17, 18). KP alterations have been identified as components in the etiology of AD and other stated neurodegenerative diseases (19–21). 3-HK and KYNA, two metabolites of TRP, have excitotoxic and neuroprotective effects on neural cells, respectively (22–24). The enzymes IDO and KMO have a pivotal role in 3-HK production by the KP, and can be induced through pro-inflammatory cytokines (25). Enhanced activity of IDO increases the KYN/TRP ratio in the cerebrospinal fluid (CSF) (22, 23, 26). A higher ratio of KYN/TRP shows the increased transformation of TRP to KYN. In this condition KMO can accelerate 3-HK production, and 3-HK will be produced faster than KYNA. So, increased levels of 3-HK can lead to increased levels of its down-stream metabolites, like QUIN which is neurotoxic (27). Therefore, this increased ratio of KYN/TRP can be related to the reduction in cognitive performance (28).

PD is another debilitating yet common neurodegenerative disorder (29, 30), while HD is an autosomal dominant neurodegenerative disease that is characterized by cognitive, psychiatric, and metabolic dysfunctions (31). Similar to AD mechanisms, high concentration of 3-HK can cause cell death by producing free radicals in PD and HD (20, 24, 26). Although PD and HD present with similar symptoms, there are differences in the underlying mechanisms between these two diseases. For example, the level of 3-HK has been increased in the putamen and substantia nigra pars compacta (SNc) in PD patients, and, conversely, increased in the striatum and cortex of patients with HD (20, 24).

Finally, it can be concluded that any up-regulation of the KP presumably brings about some degree of damage to the surrounding tissue due to excess production of reactive oxygen species (ROS) and neurotoxic metabolites. This pathway may play a vital role in the pathogenesis of various neuroinflammatory diseases, such as AD (32), PD, and HD (25, 33, 34).

In this systematic review and meta-analysis, the available data on the CSF, serum, and plasma concentrations of metabolites of the KP such as KYN, TRP, 3-HK, QUIN, and KYNA are assessed in AD, PD, and HD patients compared to a control group. Furthermore, we test the hypothesis that patients’ symptomatic state, age, and duration of illness exert an effect on KP metabolite concentrations and evaluate other potential confounders (gender, MMS score, and year of publication).

## Method

This review adheres to the Preferred Reporting Items for Systematic Review and Meta-Analysis (PRISMA) guidelines (35). In this article, standardized mean difference (SMD) measure is employed as summary statistic in the meta-analysis when all the included papers evaluate the same metabolites, however; it is assessed in different ways. The SMD states a size for intervention effect in each study and that is relative to variability between participants in the result measurements seen in that paper (36). In the present meta-analysis, SMD has been employed for calculating differences of mean levels of TRP, KYN, KYNA, 3-HK, AA and 5HIAA between cases who had or did not have AD, PD, or HD in CSF, blood, temporal cortex or hippocampus tissue. The authors have calculated SMD and also corresponding 95% CIs of CSF, plasma/serum, and temporal cortex or hippocampus tissue levels based on the mean, SD, and sample size. The authors have also used range and median to estimate mean and SD if necessary (37). 95% CI was calculated from SD *via* the following formula: 95% CI = mean ± 1.96 SD. SD was obtained from 75th and 25th percentiles based on the formula as follows: SD = Norm IQR = (P75–P25) ×0.7413 (IQR: inter-quartile range; P75: 75th percentile; P25: 25th percentile). In addition, this current study has systematically reviewed QUIN levels in 5 studies. Fasting status has been stated in the studies that have reported this status.

### Search strategy

The present study is a systematic review and meta-analysis to assess the concentration of KP metabolites, including KYN, TRP, 3-HK, QUIN, and KYNA, in AD, PD, and HD patients compared to a control group. This study was conducted by reviewing the original documentations published before or during 2021. We used databases including PubMed/Medline, Scopus, Google Scholar, Web of Science, and EMBASE. The keywords “kynurenine pathway” were combined with either “Parkinson Disease” or “Huntington Disease” or “Alzheimer Disease”, and the search was limited to English language documents.

### Inclusion and exclusion criteria

Our criteria for inclusion of articles in this meta-analysis was reporting the concentration of KP metabolites in AD, PD, or HD patients compared to a control group. The process of choosing articles was begun with, two independent researchers assessing the articles by their titles and abstracts and removing duplicated documentations in order to determine and select the relevant topics. Following this, the full text of selected articles was independently reviewed by two authors. Studies without adequate data and non-human studies were excluded, for a grand total of 30 observational studies considered by this review.

### Quality assessment

We used the Newcastle-Ottawa Quality Assessment Scale (NOS) to assess the quality of the included studies (37). This scale consists of 8 items that assess and evaluate the quality of applicable studies. The components assess selection, comparability, and outcome, based on the Ottawa checklist for cross-sectional studies. Based on the final score on the NOS checklist, studies can be classified as very good quality (9-10 score), good quality (7-8 score), satisfactory quality (5-6 score), and unsatisfactory quality (0-4 score) (see **Supplementary Table 1**).

### Data extraction

Included studies were screened and evaluated *via* NOS by two independent authors, and any disagreement was resolved by the third author. The following data were extracted for final analysis: sample size, mean age of patients, blood and CSF levels of TRP, KYN, KYNA, AA, and 3-HK in AD, PD, and HD. This study was approved by the Iranian National Committee for Ethics in Biomedical Sciences (Code of Ethics: IR.SBMU.RETECH.REC.1399.993).

### Statistical analysis

This meta-analysis was performed to compare the concentration of KP metabolites including KYN, TRP, 3-HK, and KYNA in AD, PD, and HD patients compared to a control group using Stata version 15 (Stata Corp, College Station, TX, USA). After data extraction, meta-analysis has been performed if there were adequate data for a metabolite. Data for QUIN did not meet the required criteria for meta-analysis, so we gather all information on **Tables 1**, **3** to be systematically reviewed and stated in the result section. Standardized mean difference (SMD) between patients and the control group were used as unit of analysis for the KP metabolites. We used the cut-off values set by Cohen for interpretation of small, medium, and large effect sizes (0.2, 0.5, and 0.8, respectively) (58). Analyses were done using the random effects model. Heterogeneity was assessed by I^2^ statistics and values greater than 50% were considered moderate to high heterogeneity. We also performed meta-regression when there were enough number of studies to examine Mini-Mental State Exam (MMSE) as a potential effect modifier. Publication bias was visually inspected though funnel plots and quantitatively investigated using Egger’s regression test.

**Table 1:** Baseline characteristics of included AD studies.

Author, Year	N(HC/AD)	Age (mean ± SD)(HC/AD)	Male (N.)(HC/AD)	Disease duration (SD)	MMS scores	Materials	Metabolites	Fasting status	Key findings
González-Sánchez, 2020 (38)	(23/20)	64.7 (10.8)/73.3 (7.2)	15/7	4 (1.6)	16	CSF	TRP, KYNA		Tryptophan levels higher in AD group Kynurenic acid levels significantly higher in AD group
González-Sánchez, 2020 (38)	(20/9)					Serum	TRP, KYNA		Tryptophan levels lower in AD group Kynurenic acid levels higher in AD group
Sorgdrager, 2019 (21)	(38/33)	71.3 (10.7)/73.7 (6.0)	18/15	3	16.2	Serum	KYN, TRP,3-HK		Kynurenine levels higher in AD group Tryptophan levels lower in AD group 3-HK lower in AD group
Sorgdrager, 2019 (21)	(35/31)	71.3 (10.7)/73.7 (6)	18/15			CSF	KYN, TRP		Kynurenine levels higher in AD group Tryptophan levels higher in AD group
Giil, 2017 (19)	(42/42)	78.55 (6.84)/78.46 (6.34)				Plasma	KYN, TRP, KYNA, AA, 3-HK, QUIN		Kynurenine levels significantly lower in AD group Tryptophan levels significantly lower in AD group Kynurenic acid levels lower in AD group Anthranilic acid significantly lower in AD group 3-HK lower in AD group QUIN levels significantly lower compared to controls
Daouk, 2013 (39)	(38/40)	69.5/69	13/10	3.7	23	CSF	5-HIAA		5-HIAA levels higher in AD group
Oxenkrug, 2017 (40)	(24/20)	Range: 60 to 75	12/8		21.6	Serum	KYN, TRP, KYNA, AA, 3-HK		Kynurenine levels higher in AD group Tryptophan levels lower in AD group Kynurenic acid levels lower in AD group Anthranilic acid significantly lower in AD group 3-HK higher in AD group
Gulaj, 2010 (22)	(18/34)	76.17 (7.30)/78.82 (5.66)	5/10			Plasma	KYN, TRP, KYNA, AA, QUIN	In AD patients and healthy voluntaries blood was taken in the morning between 8.00-9.00 a.m.	Kynurenine levels higher in AD group Tryptophan levels significantly lower in AD group Kynurenic acid levels significantly lower in AD group Anthranilic acid significantly lower in AD group QUIN levels significantly higher in AD group
Hartai, 2007 (41)	(31/28)	73 (8.3)/77 (6.3)	10/6		21	Serum	KYN, KYNA	The blood samples were taken between 9 and 11 am, separated immediately and kept at 80 -C until measurements. None of the subjects were on a special diet.	Kynurenine levels higher in AD group Kynurenic acid levels significantly lower in AD group
Widner, 2000 (28)	(20/21)	73.4 (7.4)/74.4 (5.4)	10/6			Serum	KYN, TRP		Kynurenine levels higher in AD group Tryptophan levels lower in AD group
Baran, 1999 (42)	(13/11)	80.1 (2.4)/81 (1.9)	7/2			Postmortem	KYN, KYNA,3-HK		Kynurenine levels lower in AD group Kynurenic acid levels higher in AD group 3-HK lower in AD group
Bonaccorso, 1998 (43)	(15/15)	75.6 (9.1)/78.4 (10.3)	8/3			Plasma	TRP	blood samples were taken at 7:45 ( ± 30 min) after an overnight fast.	Tryptophan levels lower in AD group
Fekkes, 1998 (44)	(17/14)	70.1 (1.3)/73.6 (6.3)	17/4			Plasma	TRP	blood (4ml) was drawn between 11.00 and 12:00 a.m.	Tryptophan levels lower in AD group
Tohgi, 1995 (45)	(10/15)	69 (6)/68 (6)		3.4 (2.1)	17.1	CSF	KYN, TRP,5-HIAA,3-HK	Lumbar CSF was obtained between 9 and 10 o’clock in the morning, after overnight bedrest and fasting	Kynurenine levels lower in AD group Tryptophan levels lower in AD group 5-HIAA levels higher in AD group 3-HK significantly lower in AD group
Tohgi, 1992 (46)	(10/14)	68.5 (6.1)/68.4 (10.1)		3.7 (3.5)	12.9	CSF	KYN, TRP,5-HIAA, 3-HK	Lumbar CSF was obtained with the patients in the lateral decubitus position between 09.00 and 10.00 h after overnight bed-rest and fasting.	Kynurenine levels lower in AD group Tryptophan levels lower in AD group 5-HIAA levels lower in AD group 3-HK significantly lower in AD group
Beal, 1992 (47)	(21/13)	63 (3)/-	11/-			Postmortem	KYN, KYNA, 3-HK		Kynurenine levels higher in AD group Kynurenic acid levels lower in AD group 3-HK higher in AD group
Pearson, 1992 (48)	(12/12)	76(10)/79(7)	7/3			Postmortem	3-HK		3-HK higher in AD group
Baker, 1989 (49)	(13/12)	82.5(7.1)/80.6(7.3)	4/4			Postmortem	5-HIAA		5-HIAA levels significantly lower in AD group
Kay, 1986 (50)	(14/30)	64.7 (3.1)/66.8 (1.7)			16.2	CSF	5-HIAA	Fasted	5-HIAA levels higher in AD group

### Publication bias

The authors assessed publication bias of the chosen papers by Egger’s funnel plot and Begg’s test (59, 60), where *P*< 0.05 represented a significant publication bias (**Figure 4**).

Authors performed a linear regression analysis for publication bias that included both intercept and slope parameters. It was calculated based on the following equation:


(1) yi=α+βxi+ϵi

i = 1… r (r =the number of studies), yi = standardized estimate, xi = precision of studies, ϵi = error terms

## Results

### Study selection and characteristics

**Figure 1:**
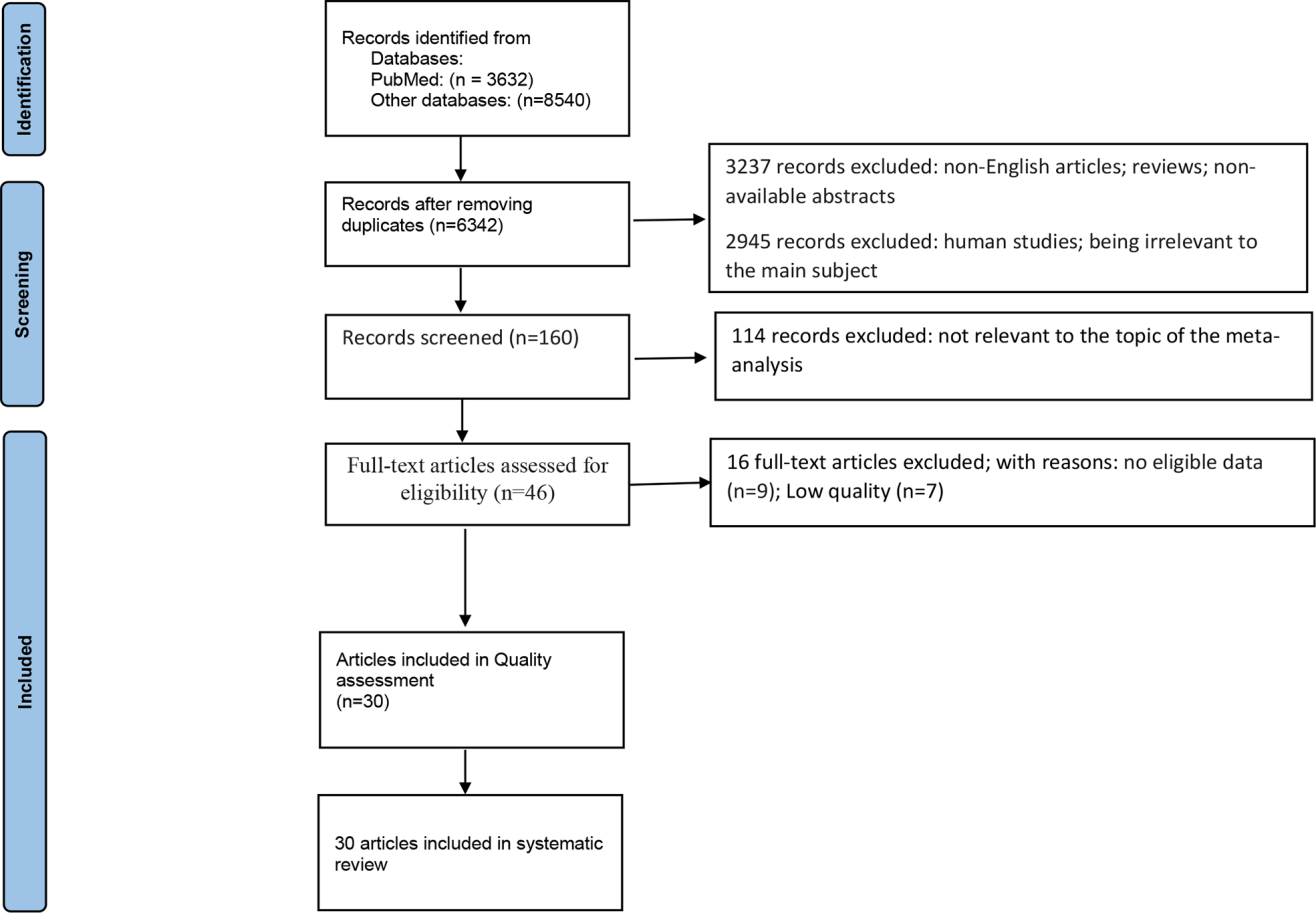
PRISMA 2020 flow diagram for systematic reviews which included searches of databases.

The current study has been run based on PRISMA checklist (61). Initially, 12172 individual studies were found through strategic search. 5830 articles were eliminated after the abstracts were screened (3237 records excluded for reasons such as: non-English articles; reviews; non-available abstracts, and 2945 removed for not being relevant to the main subject). Of the remaining 160 papers, 114 were removed because of being irrelevant to this meta-analysis. The full texts of the 46 remaining articles were fully assessed, and 16 more studies were excluded due to unclear or insufficient data (n= 9) and low quality (n=7). After full-text screening of the remaining articles, 30 articles were eligible and included in the meta-analysis (19, 21, 22, 24, 28, 31, 38–48, 50–57, 62–66) (**Figure 1**). The included studies comprised 101 comparisons of KP metabolites between patients and controls, 689 patients with Alzheimer’s disease, Parkinson’s disease, or Huntington’s disease, and 774 controls. The median sample size of the included studies was 36 participants (range: 22-242). The sample source for 33 comparisons was CSF, 59 blood, and nine brain tissues. The median age of the participants was 62.6 (range: 34-81). The results of each disease are reported as follows (**Tables 1**–**3**).

**Table 2:** Baseline characteristics of included PD studies.

Author, Year	N(HC/PD)	Age (mean ± SD)(HC/PD)	Male (N.)(HC/PD)	Materials	Metabolites	Fasting status	Key findings
Sorgdrager, 2019 (21)	(35/26)	71.3 (10.7)/73.4 (6.5)	18/20	CSF	TRP, KYN, 3-HK		Tryptophan levels lower in PD group Kynurenine levels higher in PD group 3-HK levels lower in PD group
Sorgdrager, 2019 (21)	(38/32)	71.3 (10.7)/73.4 (6.5)	18/20	Serum	TRP, KYN, 3-HK		Tryptophan levels significantly lower in PD group Kynurenine levels higher in PD group 3-HK levels lower in PD group
Lwaoka, 2019 (65)	(13/20)	69 (16.7)/69 (6.4)	9/9	CSF	TRP, KYN, KYNA,3-HK	CSF samples were obtained from participants by lumbar puncture between 09:00 and 10:00 AM after several minutes of left-sided bedrest and before breakfast.	Tryptophan levels higher in PD group Kynurenine levels significantly higher in PD group Kynurenic acid levels lower in PD group 3-HK levels significantly higher in PD group
Havelund, 2017 (66)	(14/8)	55.8 (11.6)/62.6 (6.6)	8/5	CSF	KYN, KYNA,AA,3-HK	Plasma and cerebrospinal fluid samples were collected after overnight fasting and 1-2 hours after intake of L-DOPA or other anti-Parkinson medication.	Kynurenine levels higher in PD group Kynurenic acid levels higher in PD group ANT levels higher in PD group 3-HK levels lower in PD group
Havelund, 2017 (66)	(14/8)	55.8 (11.6)/62.6 (6.6)	8/5	Plasma	KYN, KYNA, AA, 3-HK		Kynurenine levels lower in PD group Kynurenic acid levels lower in PD group ANT levels higher in PD group 3-HK levels lower in PD group
Oxenkrug, 2017 (40)	(24/18)	Range: 50 to 74	12/11	Serum	TRP, KYN, KYNA, AA	Overnight fasting blood samples were collected	Tryptophan levels significantly lower in PD group Kynurenine levels significantly higher in PD group Kynurenic acid levels significantly higher in PD group ANT levels significantly higher in PD group
Chang, 2017 (51)	(82/82)	62.83 (12.66)/62.87 (12.49)	50/50	Plasma	TRP, KYN, KYNA, AA, 3-HK, QUIN		Tryptophan levels significantly lower in PD group Kynurenine levels significantly lower in PD group Kynurenic acid levels significantly lower in PD group ANT levels lower in PD group 3-HK levels higher in PD group QUIN levels significantly higher in PD group
Hartai, 2005 (24)	(17/19)	67.8 (12.4)/69.1 (10.4)	8/11	Plasma	KYNA		Kynurenic acid levels lower in PD group
Ruiz, 1995 (54)	(15/23)	39.0(17.8)/58(10.0)	-/13	CSF	5-HIAA	Lumbar CSF after overnight bed-rest and fasting.	5-HIAA levels higher in PD group
Molina, 1997 (64)	(45/31)	57.8 (15.4)/62.6 (8.7)	18/11	CSF	TRP		Tryptophan levels higher in PD group
Molina, 1997 (64)	(45/31)	57.8 (15.4)/62.6 (12.5)	18/11	Plasma	TRP	Blood samples for metabolomics analysis were collected from subjects who were asked to be on fasting overnight for 8 h.	Tryptophan levels significantly lower in PD group
Tohgi, 1993 (63)	(10/16)	65.5 (6.2)/62.6 (8.7)		CSF	TRP, KYN,5-HIAA,3-HK	Lumbar CSF after overnight bed-rest and fasting.	Tryptophan levels lower in PD group Kynurenine levels significantly lower in PD group 5-HIAA significantly lower in PD group 3-HK levels significantly lower in PD group
Tohgi, 1993 (62)	(16/16)	63.9 (8.1)/62.6 (8.7)		CSF	TRP, KYN, 5-HIAA,3-HK	Lumbar CSF after overnight bed-rest and fasting.	Tryptophan levels lower in PD group Kynurenine levels significantly lower in PD group 5-HIAA levels lower in PD group 3-HK levels significantly lower in PD group
Beal, 1992 (47)	(18/11)	69(3)/66(4)	8/8	Postmortem	3-HK		3-HK levels lower in PD groups

**Table 3:** Baseline characteristics of included HD studies.

Author, Year	N(HC/HD)	Age (mean ± SD)(HC/HD)	Male (N.) (HC/HD)	Materials	Metabolites	Fasting status	Key findings
Chang, 2017 (51)	(47/22)	65.32 (8.62)/43.77 (10.88)	23/15	Plasma	TRP, KYN, 3-HANA, QUIN		Tryptophan levels higher in HD group KYN levels significantly lower in HD group 3-HANA levels significantly lower in HD group QUIN levels significantly lower in HD group
Forrest, 2010 (52)	(11/14)	52.11 (4.34)/48.95 (1.58)	2/5	Blood	TRP, KYN, 3-HANA		Tryptophan levels lower in HD group KYN levels lower in HD group 3-HANA levels higher in HD group
Christofides, 2006 (53)	(15/11)	44.6 (9.0)/61.6 (7.5)	4/3	Blood	TRP	All subjects were fasted overnight	Tryptophan levels higher in HD group
Ruiz, 1995 (54)	(15/20)	39(17.8)/36.1(14.9)	-/13	Blood	TRP, KYN, 3-HANA	Lumbar CSF after overnight bed-rest and fasting.	Tryptophan levels higher in HD group KYN levels significantly lower in HD group 3-HANA levels significantly lower in HD group
Beal, 1992 (47)	(23/45)	66 (2)/57 (2)	13/27	Postmortem	TRP, KYNA, 3-HK		Tryptophan levels higher in HD group KYNA levels significantly lower in HD group 3-HK levels higher in HD group
Belendiuk, 1980 (55)	(51/25)	34.0 (11.5)/47.4 (11.3)	30/16	Blood	TRP,		Tryptophan levels significantly lower in HD group
Guidetti, 2004 (56)	(17/3)	61.0 (3.3)/72.1 (5.2)	–	Postmortem	KYNA, 3-HK, QUIN		KYNA levels significantly higher in HD group 3-HK levels lower in HD group QUIN levels significantly higher in HD group in low grade stage No significant changes in QUIN was observed in advanced stage
Jauch, 1995 (57)	(17/17)	59 (19)/63 (12)	(15/9)	Postmortem	KYNA		KYNA levels lower in HD group
Pearson S., 1992 (48)	(21/22)	63 (14)/57 (13)	(17/11)	Postmortem	3-HK		3-HK levels significantly higher in HD group

### Alzheimer’s disease

A total of 17 included articles studied AD (19, 21, 22, 28, 38–48, 50). They contained 698 participants (362 patients and 346 controls) and generally 47 comparisons of KP metabolites Three comparisons were performed in the temporal cortex or hippocampus tissue, 14 in the CSF, and 30 in the blood (serum or plasma). **Table 1** indicates the characteristics of the included studies and their data on the investigated metabolites.

Blood levels of TRP were significantly lower in the patients than the healthy controls (SMD=-0.68, 95% CI=-0.97 to -0.40, p=0.000, I2 = 41.8%, k=8, n=382), which was a medium difference according to Cohen (58). Blood levels of KYN (SMD=0.62, 95% CI=-0.18 to 1.43, p=0.127, I2 = 92%, k=6, n=351), KYNA (SMD=-1.03, 95% CI=-2.19 to 0.13, p=0.083, I2 = 94.1%, k=5, n=268), AA (SMD=-0.34, 95% CI=-0.72 to 0.05, p=0.084, I2 = 35.6%, k=3, n=180), and 3-HK (SMD=-0.05, 95% CI=-0.58 to 0.49, p=0.858, I2 = 71.1%, k=3, n=199) were not significantly different between patients and controls.

CSF levels of 3-HK were lower in patients than in healthy controls (SMD=-1.28, 95% CI=-2.35 to -0.20, p=0.020, I2 = 81.7%, k=3, n=115). The 3-HK difference between patients and controls was large according to Cohen (58). No significant differences were found between patients and controls in the CSF levels of TRP (SMD= -0.34, 95% CI=-0.97 to 0.29, p=0.284, I2 = 71%, k=4, n=158), KYN (SMD=-0.62, 95% CI=-1.70 to 0.45, p=0.254, I2 = 84%, k=3, n=115), and 5HIAA (SMD=0.36, 95% CI=-0.13 to 0.84, p=0.151, I2 = 53.9%, k=4, n=171). Also, the trend of an increased KYN to TRP ratio in the blood of patients compared to controls was not significant (SMD=0.46, 95% CI= -0.01 to 0.92, p=0.053, I2 = 72.9%, k=5, n=292).

Levels of 3-HK in the temporal cortex or the hippocampus of AD patients were not significantly different from healthy controls (SMD=0.10, 95% CI=-0.39 to 0.60, p=0.681, I2 = 19.4%, k=3, n=72).

Three studies reported the level of QUIN in AD patients. First study conducted by Gulaj et al. (22), reported that a marked increase in QUIN blood level in AD patients compared to controls and inverse correlation between cognitive function tests and QUIN level (334 VS. 192, P value< 0.05). In contrast, a study conducted by Giil et al. (19), reported that blood level of QUIN was significantly lower in AD patients compared to controls (465 ± 230 VS 565 ± 370, P value< 0.05).

Six studies reported data regarding fasting status of participants and in all of them participants entered in fasting states (22, 41, 43–46).

The subgroup analysis showed significantly lower levels of TRP in serum (SMD=-0.41, 95% CI=-0.71 to -0.12, p=0.006, I2 = 0.0%, k=4, n=203), and also plasma (SMD=-0.99, 95% CI=-1.45 to -0.52, p=0.000, I2 = 53.5%, k=4, n=243) of patients compared to the controls. Thus, the difference between patients and controls in the TRP levels was small in serum and large in plasma. We did not conduct subgroup analysis for other metabolites because of the limited number of studies.

Meta-regression analysis did not show a significant association between average MMSE scores and blood levels of KYN (p=0.788). There was also no significant association between MMSE scores and the CSF levels of 5-HIAA (p=0.129).

**Figure 2:**
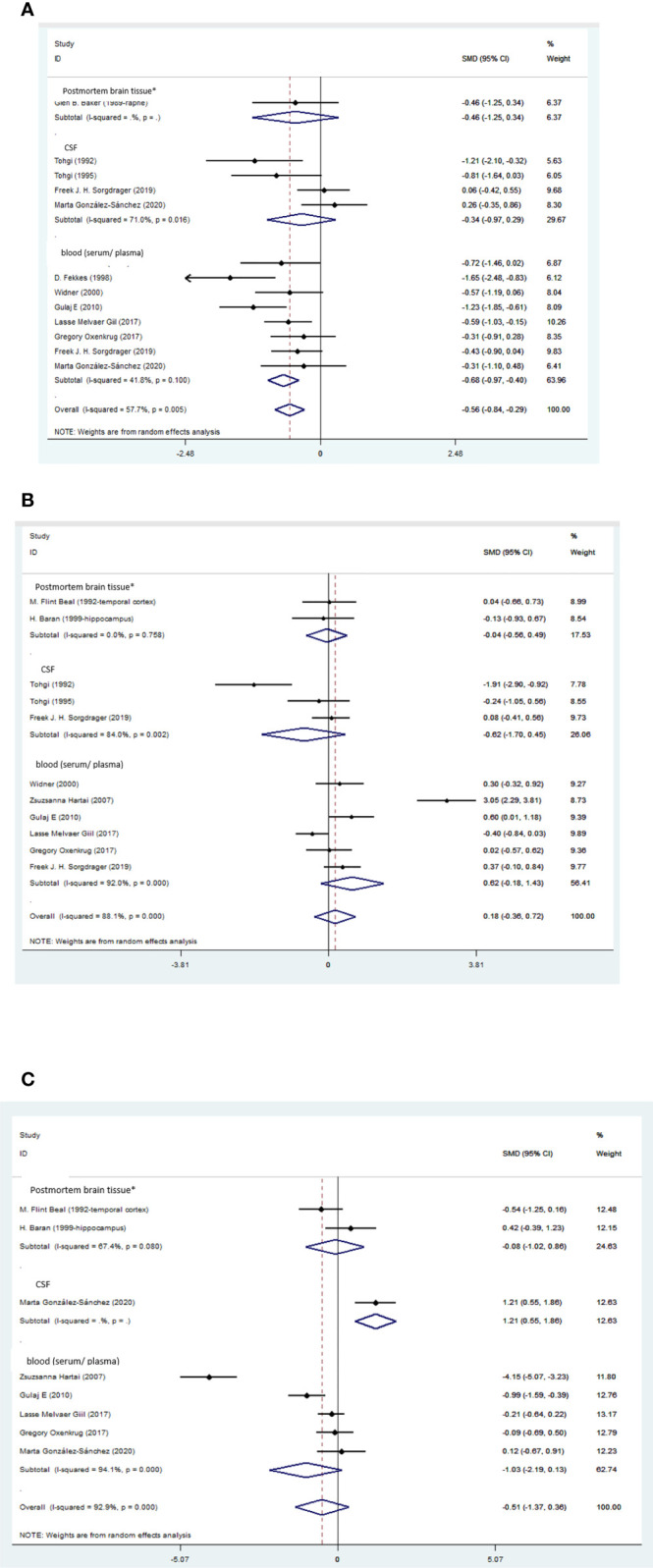
**(A)** Forest plot of the levels of TRP in AD patients. In this plot, the squares are applied to show the mean effect estimate of each paper along with their 95% CI. The size of each square is considered proportional to the weight of the parameter in the meta-analysis, and is also demonstrated in a separate column. *temporal cortex or hippocampus. **(B)** Forest plot of the levels of KYN in AD patients. In this plot, the squares are applied to show the mean effect estimate of each paper along with their 95% CI. The size of each square is considered proportional to the weight of the parameter in the meta-analysis, and is also demonstrated in a separate column. *temporal cortex or hippocampus. **(C)**. Forest plot of the levels of KYNA in AD patients. In this plot, the squares are applied to show the mean effect estimate of each paper along with their 95% CI. The size of each square is considered proportional to the weight of the parameter in the meta-analysis, and is also demonstrated in a separate column. *temporal cortex or hippocampus.

**Figure 3:**
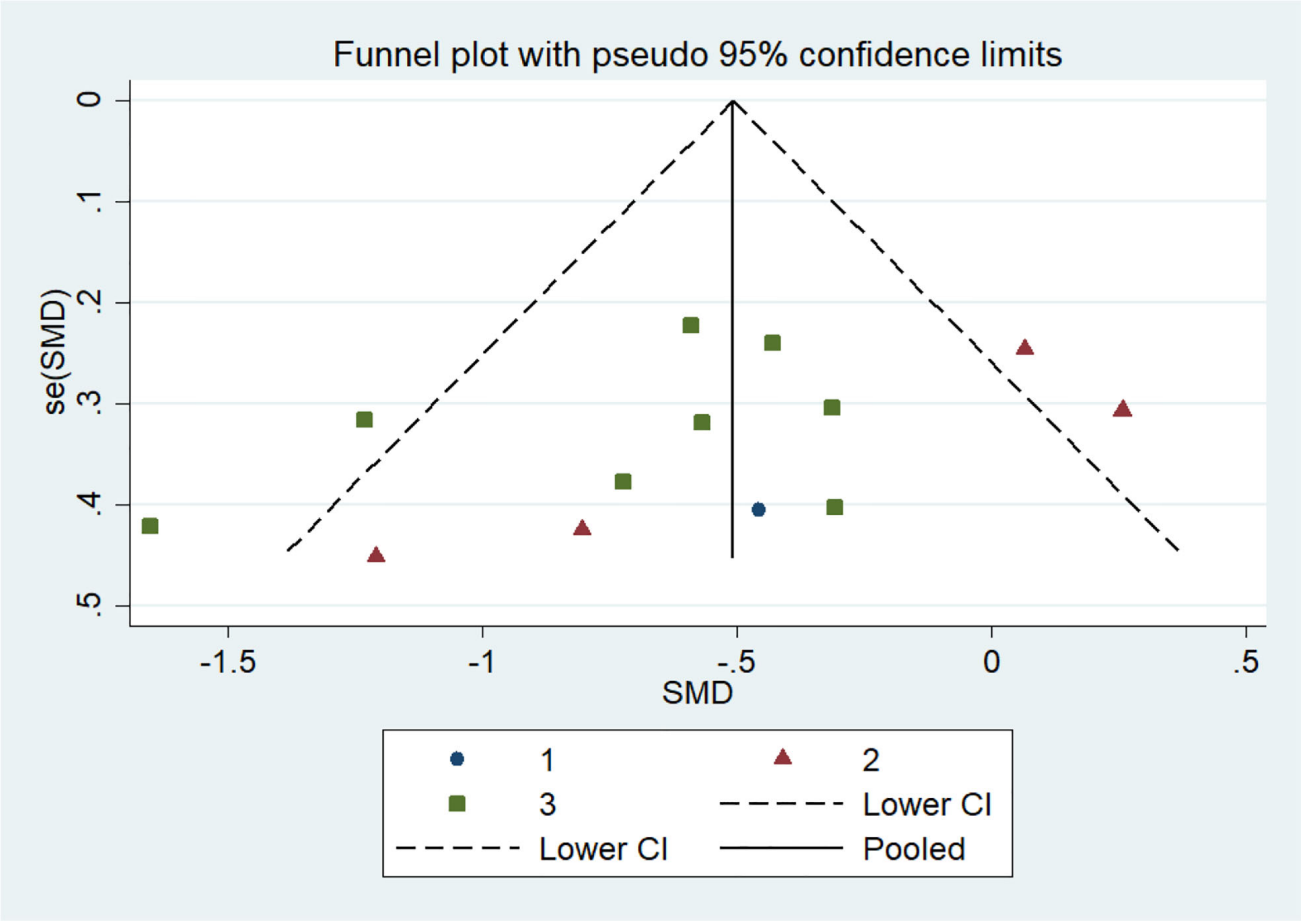
Funnel plot for publication bias with pseudo 95% confidence limits for AD based on TRP.

**Figure 4:**
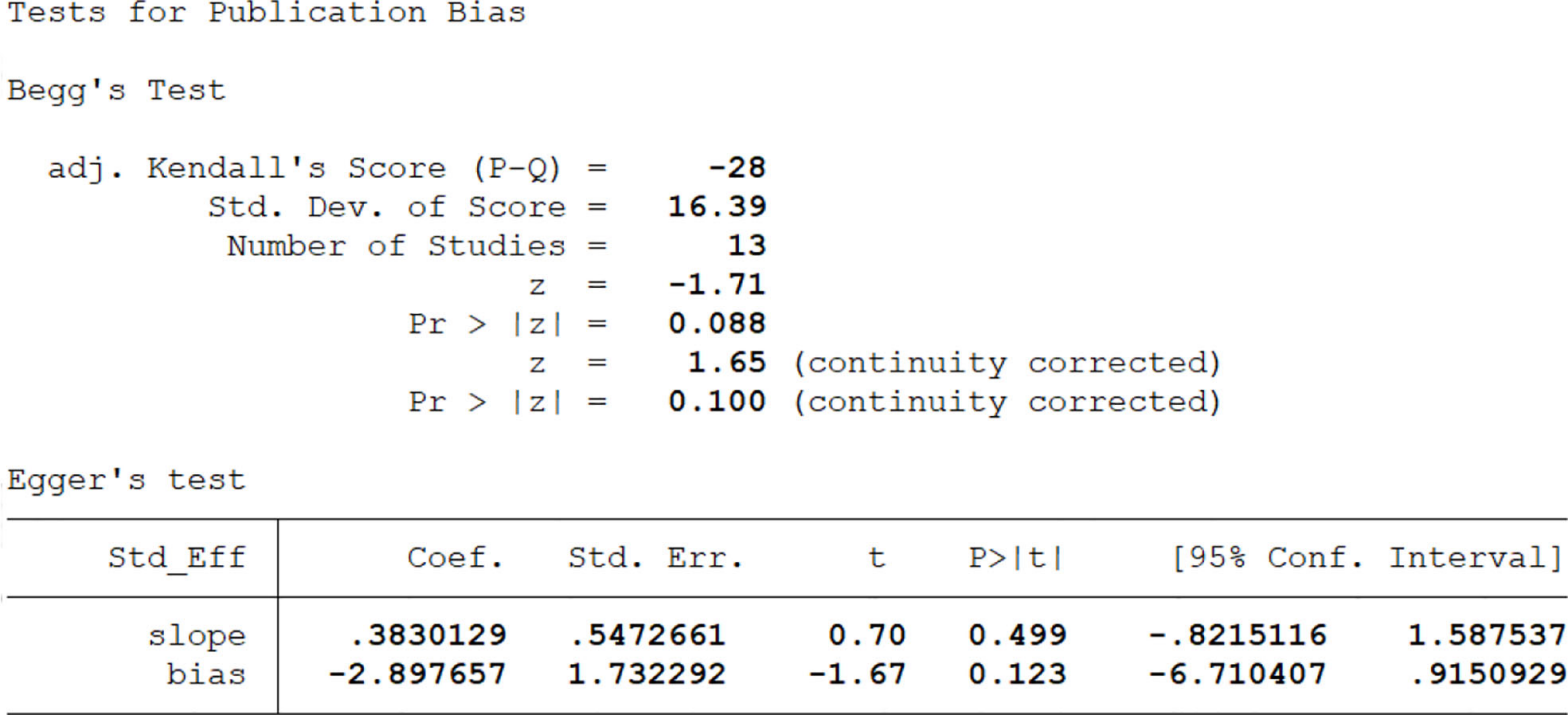
Begg and Egger’s test for publication bias with pseudo 95% confidence limits for AD.

We performed Egger’s test to assess publication bias, which was not found to be significant for TRP (p=0.499). The funnel plot is shown in the supplementary material. (**Table 4**, **Figures 2A-C**; **3**, **4**, **Supplementary Figures 3D-F**).

**Table 4:** Statistical analysis of reviewed studies for AD.

Metabolite	Materials	Number of studies	SMD	95%CI	I^2^(%)	P-value for heterogenicity
TRP	CSF	4	-0.34	(-0.97, 0.29)	71.0	0.016
	Blood (serum/plasma)	8	-0.68	(-0.97, 0.40)	41.8	0.100
KYN	CSF	3	-0.62	(-1.70, 0.45)	84.0	0.002
	Blood (serum/plasma)	6	0.62	(-0.18, 1.43)	92.0	0.000
KYNA	Blood (serum/plasma)	5	-1.03	(-2.19, 0.13)	94.1	0.000
KYN/TRP	Blood (serum/plasma)	5	0.46	(-0.01, 0.92)	72.9	0.005
AA	Blood (serum/plasma)	3	-0.34	(-0.72, 0.05)	35.6	0.211
5HIAA	CSF	4	0.36	(-0.13, 0.84)	53.9	0.089
3-HK	Temporal cortex/Hippocampi	3	0.10	(-0.39, 0.60)	19.4	0.289
	CSF	3	-1.28	(-2.35, -0.20)	81.7	0.004
	Blood (serum/plasma)	3	-0.05	(-0.58, 0.49)	71.1	0.031

### Parkinson’s disease

Ten studies were included (21, 24, 40, 51, 54, 62–66) and were comprised of 37 comparisons and 539 participants (265 patients and 274 controls). We conducted nineteen comparisons in CSF and 18 in blood. **Table 2** indicates the characteristics of the included studies and their data on the investigated metabolites.

Blood levels of TRP were significantly lower in patients than controls (SMD=-0.77, 95% CI=-1.24 to -0.30, p=0.001, I2 = 74.9%, k=4, n=352), which was a medium difference (58). There were no significant differences between patients and controls in the blood levels of KYN (SMD=0.28, 95% CI=-0.44 to 0.99, p=0.447, I2 = 86.2%, k=4, n=298), KYNA (SMD=-0.14, 95% CI=-1.07 to 0.80, p=0.775, I2 = 89.6%, k=4, n=264), AA (SMD=0.38, 95% CI=-0.40 to 1.16, p=0.336, I2 = 80.9%, k=3, n=228), or 3-HK (SMD=-0.01, 95% CI=-0.41 to 0.39, p=0.965, I2 = 48.1%, k=3, n=256).

**Figure 5:**
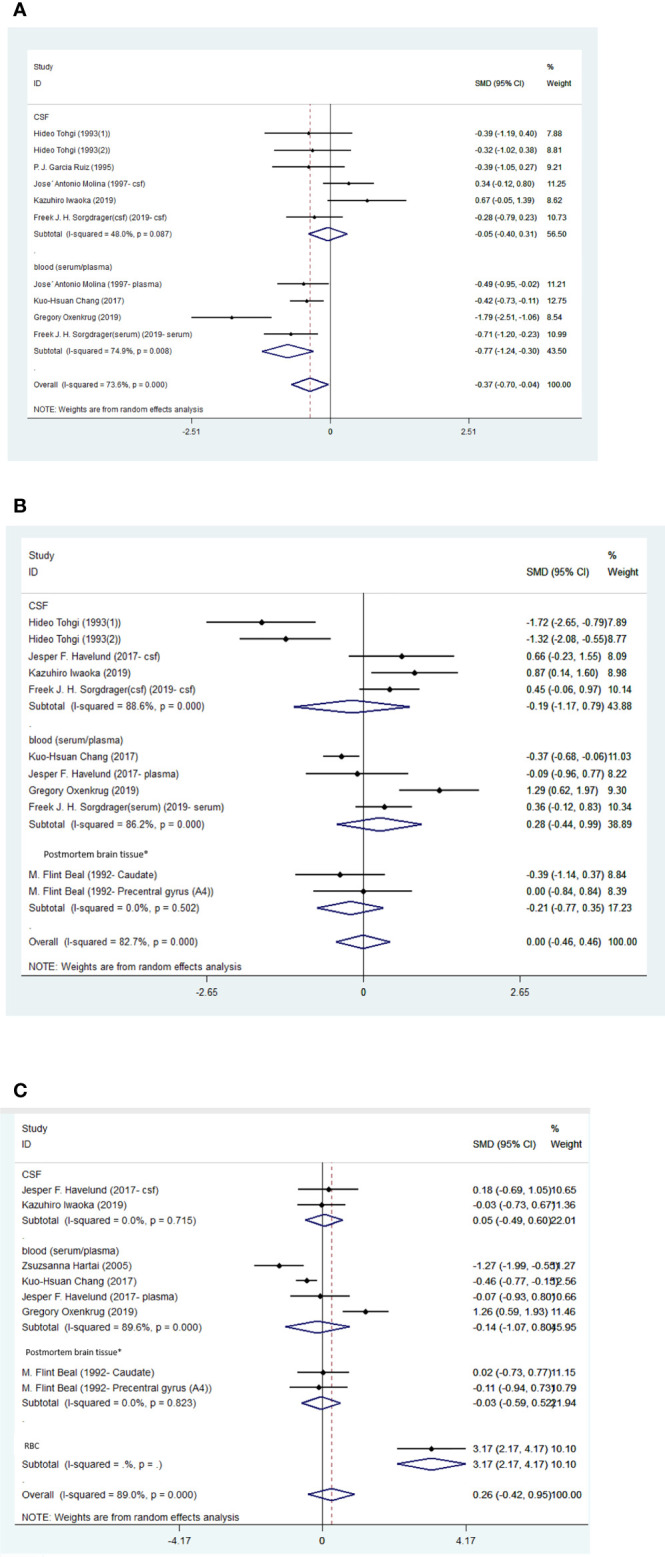
**(A)** Forest plot of the levels of TRP in PD patients. In this plot, the squares are applied to show the mean effect estimate of each paper along with their 95% CI. The size of each square is considered proportional to the weight of the parameter in the meta-analysis, and is also demonstrated in a separate column. **(B)**. Forest plot of the levels of KYN in PD patients. In this plot, the squares are applied to show the mean effect estimate of each paper along with their 95% CI. The size of each square is considered proportional to the weight of the parameter in the meta-analysis, and is also demonstrated in a separate column. *caudate nucleus or precentral gyrus. **(C)** Forest plot of the levels of KYNA in PD patients. In this plot, the squares are applied to show the mean effect estimate of each paper along with their 95% CI. The size of each square is considered proportional to the weight of the parameter in the meta-analysis, and is also demonstrated in a separate column. *caudate nucleus or precentral gyrus.

There were no significant differences between patients and controls in the CSF levels of TRP (SMD=-0.05, 95% CI=-0.40 to 0.31, p=0.795, I2 = 48%, k=6, n=263), KYN (SMD=-0.19, 95% CI=-1.17 to 0.79, p=0.705, I2 = 88.6%, k=5, n=171), 5HIAA (SMD=-0.42, 95% CI=-1.23 to 0.39, p=0.311, I2 = 72.9%, k=3, n=96), or 3-HK (SMD=-0.97, 95% CI=-2.28 to 0.34, p=0.146, I2 = 92.6%, k=5, n=171). Egger’s test did not indicate publication bias for KYN (p=0.869). The funnel-plot is shown in the supplementary material. (**Table 5**; **Figures 5A–C**; **Supplementary Figures 4D–I**).

**Table 5:** Statistical analysis of reviewed studies for PD.

Metabolite	Materials	Number of studies	SMD	95%CI	I^2^(%)	P-value for heterogenicity
TRP	CSF	6	-0.05	(-0.40, 0.31)	48.0	0.087
	Blood (serum/plasma)	4	-0.77	(-1.24, -0.30)	74.9	0.008
KYN	CSF	5	-0.19	(-1.17, 0.79)	88.6	0.000
	Blood (serum/plasma)	4	0.28	(-0.44, 0.99)	86.2	0.000
KYNA	Blood (serum/plasma)	4	-0.14	(-1.07, 0.80)	89.6	0.000
AA	Blood (serum/plasma)	3	0.38	(-0.40, 1.16)	80.9	0.005
5HIAA	CSF	3	-0.42	(-1.23, 0.39)	72.9	0.025
3-HK	CSF	5	-0.97	(-2.28, 0.34)	92.6	0.000
	Blood (serum/plasma)	3	-0.01	(-0.41, 0.39)	48.1	0.146

A study conducted by Chang et al. (51), reported significantly higher level of blood QUIN in PD patients compared to controls (0.376 ± 0.175 VS. 0.205 VS. 0.077).

Six studies reported data regarding fasting status of participants and in all of them participants entered in fasting states (40, 46, 54, 63–66).

### Huntington’s disease

A total of nine studies (31, 47, 48, 51–53, 55–57), comprising 17 comparisons and 603 participants (261 patients and 342 controls), were included. Eleven comparisons were measured in blood, and six comparisons were made according to the metabolites measured in the frontal cortex. **Table 3** shows the characteristics of the included studies and their data on the investigated metabolites.

Blood levels of TRP (SMD=-0.90, 95% CI=-1.71 to -0.10, p=0.028, I2 = 91.0%, k=5, n=369) and KYN (SMD=-0.37, 95% CI=-0.68 to -0.06, p=0.020, I2 = 28.5%, k=3, n=336) were significantly lower in patients than controls. The differences the in levels of TRP and KYN between patients and controls were respectively large and small (58). Blood levels of 3-HANA were not significantly different between patients and controls (SMD=0.32, 95% CI=-0.75 to 0.12, p=0.152, I2 = 76.2%, k=3, n=336).

**Figure 6:**
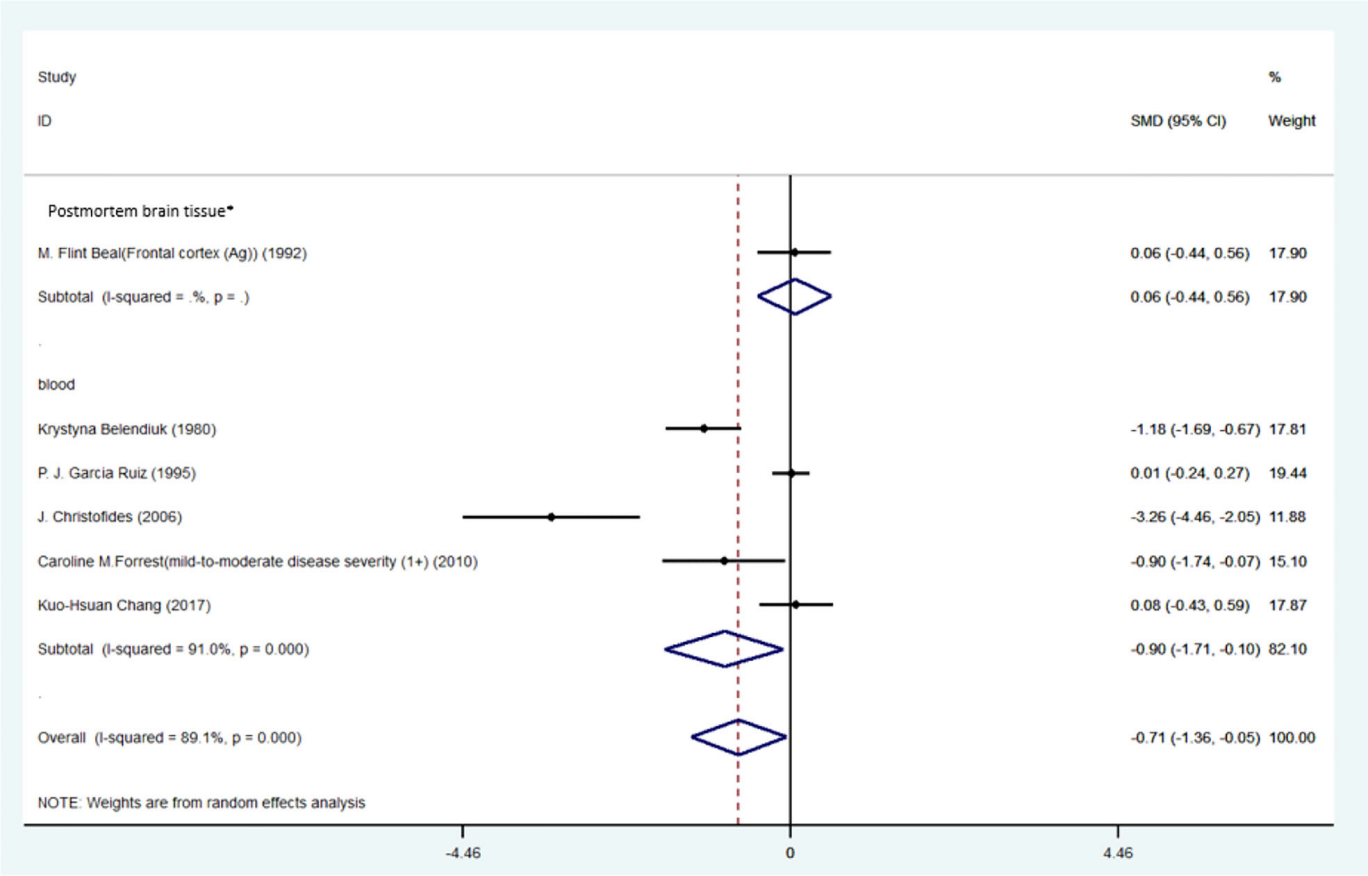
Forest plot of the levels of TRP in HD patients. In this plot, the squares are applied to show the mean effect estimate of each paper along with their 95% CI. The size of each square is considered proportional to the weight of the parameter in the meta-analysis, and is also demonstrated in a separate column. *frontal cortex.

Levels of KYNA (SMD=-0.08, 95% CI=-0.88 to 1.05, p=0.865, I2 = 78.9%, k=3, n=122) and 3-HK (SMD=0.47, 95% CI=-0.41 to 1.35, p=0.361, I2 = 76.2%, k=3, n=131) in the frontal cortex were not significantly different between patients and controls. No sign of publication bias for TRP was indicated by Egger’s test (p=0.229). The funnel-plot is shown in the supplementary material. (**Table 6**; **Figure 6A**; **Supplementary Figures 5B–G**).

**Table 6:** Statistical analysis of reviewed studies for HD.

Metabolite	Materials	Number of studies	SMD	95%CI	I^2^(%)	P-value for heterogenicity
TRP	Blood (serum/plasma)	5	-0.90	(-1.71, -0.10)	91.0	0.000
KYN	Blood (serum/plasma)	3	-0.37	(-0.68, -0.06)	28.5	0.247
KYNA	Frontal cortex	3	0.08	(-0.88, 1.05)	78.9	0.009
3-HK	Frontal cortex	3	0.47	(-0.41, 1.35)	76.2	0.015
3-HANA	Blood (serum/plasma)	3	-0.32	(-0.75, 0.12)	57.4	0.095

TRP, tryptophan; KYN, kynurenine; KYNA, kynurenic acid; AA, anthranilic acid; 5HIAA, 5-hydroxyindoleacetic acid; 3-HK, 3-hydroxykynurenine; 3-HANA, 3-hydroxyanthranilic acid; CSF, Cerebrospinal fluid; SMD, standardized mean difference; CI, confidence interval.

One study conducted by Chang et al. (51), reported that blood level of QUIN was significantly lower in HD patients compared to controls (0.205 ± 0.077 VS 0.280 ± 0.135). Second study conducted by Guidetti et al. (56), reported that in postmortem samples of low-grade HD, QUIN level was significantly higher compared to controls. In contrast in advanced grade of HD, no such changes were observed and QUIN levels were either unchanged or reduced.

Two studies reported data regarding fasting status of participants and in all of them participants entered in fasting states (53, 54).

## Discussion

### Kynurenine pathway

kynurenine pathway is one of the major route for tryptophan catabolism. In brain, tryptophan is converted to N-Formyl-L-kynurenine (NFKYN) through the action of indole amin-2, 3-dioxygenase (IDO). Thereafter, NFKYN is converted to kynurenine by kynurenine formamidase or kynurenine formylase. HYN is further catabolized to kynurenic acid (KYNA), 3-Hydroxy –L-kynurenine (3-HK), and anthranilic acid (AA). 3-HK is converted to 3-hydroxyanthranilic acid (3-HANA) the precursor of QUIN, a neurotoxic metabolite of kynurenine pathway (67). Kynurenine aminotransferases (KATs) catabolize the formation of KYNA. At high concentrations, KYNA is a competitive antagonist of N-Methyl-D-Aspartate (NMDA) receptors, while in low concentrations, is a non-competitive antagonist of nicotinic acetylcholine receptors. Therefore, KYNA is considered as a potential antioxidant (4).

More than 90% of peripheral tryptophan is converted to KYN. Kynurenine/tryptophan (K/T) ratio could be used as a marker to reflect the risk of CNS disease. In fact, in CNS disease more peripheral tryptophan is converted to kynurenine which is a precursor for production of neurotoxic metabolites such as QUIN. Moreover, increased ratio of K/T is indicative of inflammation and mental disorder in AD disease (68). These data are consistent with our results, showing significant reduction of tryptophan blood level in AD, PD, and HD diseases.

KP metabolites have various neuronal effects, they could be either neuroprotective or neurotoxic. For instance, 3-HK, 3-HAA, and 5-HAA are reported to induce neuronal death neuron cultures of rat models. These substances induce generation of ROS and subsequently lead to neurodegeneration (69, 70). QUIN another neurotoxic metabolite selectively activates NMDA receptors inducing excitation which ultimately leads to neuronal lesion in the brain of rat models (71). In contrast to neurotoxic effect of QUIN, KYNA acts as antagonists for NMDA receptors and therefore blocks neurotoxic effects of QUIN (72). Higher levels of neurotoxic metabolites together with lower levels of KYNA contributes to the progression of neurodegenerative diseases.

### Alzheimer’s disease

Our main results revealed that blood levels of (TRP) were significantly lower in AD patients compared to healthy controls (HCs) (SMD=-0.68, 95% CI=-0.97 to -0.40, p=0.000, I2 = 41.8%, k=8, n=382) (**Table 4**). In line with our results, a recent meta-analysis has assessed there to be lower levels of TRP in serum of 738 AD patients compared to 665 HCs (73). Another study on patients suffering AD reported decreased serum levels of TRP and KYN (74), and a study on 34 patients with Alzheimer-type dementia and 18 controls showed lower TRP plasma concentrations among demented individuals (75). Subjective cognitive impairment (SCI) is known to be the preclinical manifestation of AD, preceding the development of objective cognitive impairment (76–78). In opposition to our findings, a study comparing KP metabolites in patients with AD, HCs with subjective cognitive impairment (SCI), and major depression, showed that TRP serum levels were not significantly lower in AD subjects when compared SCI controls (79). However, subgroup analysis of published literature included in the present study showed significantly lower levels of TRP in serum of AD patients compared to a control population (SMD=-0.41, 95% CI=-0.71 to -0.12, p=0.006, I2 = 0.0%, k=4, n=203) (**Table 4**). This indicates that TRP could be used a biomarker to distinguish AD in its early stages. Additionally, lower TRP levels were found in other neurodegenerative diseases including multiple sclerosis and HD as well suggesting it as a screening marker for this disease (23, 80, 81).

**Figure 7:**
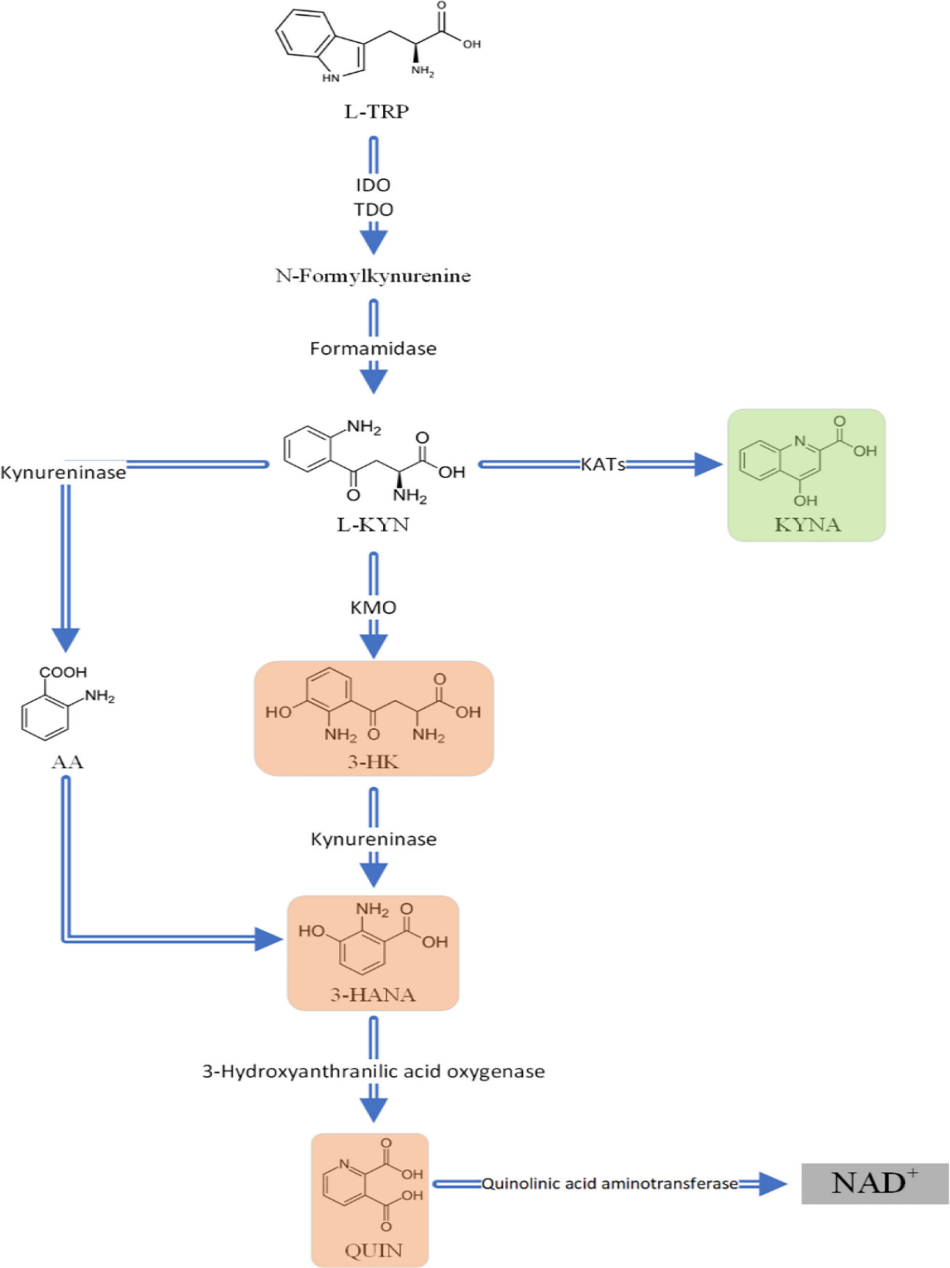
An overview of the Kynurenine pathway (KP). The metabolites highlighted in red (3,HK; 3,HANA; and QUIN) are neurotoxic and KYNA which is highlighted in green is neuroprotective. L,TRP L,Tryptophan; L,KYN L,kynurenine; KYNA Kynurenic acid; 3,HK 3,Hydroxykynurenine; 3,HANA 3,Hydroxyanthranilic acid; QUIN Quinolinic acid; AA Anthranilic acid; IDO indoleamine 2;3,dioxygenase; TDO tryptophan 2;3,dioxygenase; KATs kynurenine aminotransferases; KMO.

According to present findings, blood levels of KYN, KYNA, AA, and 3-HK levels were not significantly different between AD patients and healthy controls (**Table 4**). In a study, high-performance liquid chromatography revealed non-significantly increased levels of KYN, 3-HK and AA levels, as well (75). Likewise, our meta-regression analysis did not show a significant association between MMSE scores and blood levels of KYN. In contrast to our findings, one article reported that lower plasma levels of KYNA correlated to cognitive impairment, and therefore KYNA was suggested as a novel promising neuroprotective therapy (75). In another study, 3-HK serum levels were higher in AD when compared to patients with SCI or major depression (79). Receiver-operating characteristic (ROC) analysis of the AD group compared with the major depression and SCI groups together, indicated that with approximately 70% sensitivity and 80% specificity, 3-HK serum concentration could be a promising biomarker for AD diagnosis (79). Chatterjee and colleagues investigated the role of KP in patients suffering from preclinical AD, as defined by high neocortical amyloid-β load (NAL). Dividing patients in two groups of NAL+ and NAL-, KYN and AA serum concentrations both individually and together were proven to be significant predictors for NAL+ becoming clinical AD in women compared to men (82), though our meta-regression did not show a significant correlation. In contrast to our findings, authors also reported elevated serum levels of 3-HK in NAL+ women, and, additionally, in a cohort study measuring subjects each five years for cognitive decline and dementia, Chouraki et al. revealed higher AA concentrations in healthy individuals who later developed dementia in the follow-ups (83). These disparities highlight the necessity to further investigate KP metabolites while considering the different stages of the disease, such as SCI, mild cognitive impairment (MCI), AD, and patients with normal cognition. Sorgdrager and colleagues stated that, in using an age-by-disease interaction analysis, ageing and AD both altered KP metabolites in the same way (21).Therefore, age normalization should be considered for all investigations, and, according to Chatterjee et al., sex also plays a significant role (82). Thus, we suggest conducting more experimentally controlled investigations in order to compare metabolite and inflammation-related marker levels of AD patients and HCs simultaneously in different body fluids, including urine, plasma, serum, and CSF. Moreover, Jacobs et al. reported that decreased KYN in AD patients was inversely associated with CSF phosphorylated-tau (84). Additionally, the correlation between KP metabolites and plasma amyloid β was reported to be dependent on NAL load status (85). Thus, assessing KP metabolites and measuring biomarker evidence of neurodegeneration, including amyloid-β, total-tau, or phosphorylated-tau, could clarify the correlation between KP activation and AD biomarkers (86) (**Figure 7**).

Our study demonstrated that CSF concentrations were different from blood levels. CSF levels of 3-HK in AD patients were lower than in HCs. Previous studies demonstrated no changes in 3-HK concentrations in AD patients when compared to controls, either centrally or peripherally (21, 42, 73, 75). Notably, a recent meta-analysis involving 738 patients and 665 HCs confirmed the notion (87). Another study reported that increased total- and phosphorylated-tau in AD patients is correlated with higher levels of 3-HK (84). Since total- and phosphorylated-tau are associated with a poor prognosis of AD (88), lower levels of 3-HK in our study could represent better prognosis of our targeted population.

No significant differences were found in CSF concentrations of TRP, KYN, and 5-HIAA. In addition, there was no association between MMSE scores and the CSF levels of 5-HIAA in meta-regression analysis. Consistently with our results, Soininen et al. claimed similar CSF levels of 5-HIAA among Alzheimer-type senile dementia patients and a control group (89). Although characterized by significantly lower mean concentrations of 5-HIAA, a previous study revealed that changes in 5-HIAA CSF concentrations were nonspecific secondary to the cerebral degradation in AD (90). Morimoto et al. found a decrease in CSF levels of 5-HIAA in patients living with dementia with Lewy bodies (DLB) after the onset of clinical manifestations (91). AD and DLB are both neurodegenerative diseases but neuropathologically different. DLB is characterized by aggregation of α-synuclein neurons compared to amyloid-β plaques and tau neurofibrillary tangles found in AD pathogenesis (92, 93). The same study found that in AD patients, 5-HIAA levels become significantly lower (91). Authors suggested that the combination of 5-HIAA and total- and phosphorylated-tau in the CSF of patients could differentiate among DLB, DLB with AD, and AD. Thus, although 5-HIAA did not solely show diagnostic validity, its combination with brain degeneration-specific biomarkers could provide high diagnostic accuracy.

The KYN to TRP (K:T) ratio in serum and CSF is considered as an indicator of augmented INF- γ-associated TRP metabolism (94). Augmented serum K:T ratios have been mentioned previously in clinical AD and in individuals with MCI representing IDO activity as the first enzyme converting TRP to KYN in KP (95, 96). Chatterjee et al. showed considerably increased levels of serum K:T ratios suggesting TRP degradation *via* KP, preceding cognitive impairment (82). A study on 58 participants with normal cognition, 396 MCI, and 112 AD subjects concluded that in addition to the inflammatory cascade triggered by increased KYN levels, the higher the K:T ratios, the lower the memory scores and functional independencies (97). However, our findings showed that although a higher K: T ratio was found in the CSF of AD patients compared to controls, it was not significant. Further evaluation is essential to clarify the relevance of the K/T ratio in AD patients, investigate its generalizability, and determine the effect of IDO inhibition in disease progress.

Jacobs and colleagues highlighted a disease-independent relation between KYN, 3-HK, AA, K/T and 3-HK/KYN in both plasma and CSF samples from AD patients and matched-controls, proving that plasma levels could promisingly imply CSF concentrations and prevent unnecessary lumbar punctures (84). The results of our study did not follow the same correlation between CSF and plasma concentrations. Since the contradicting correlations have an impact on the use and regulation of invasive interventions, this discrepancy is of high importance to be assessed by further restricted studies.

### Parkinson’s disease

Impaired KP metabolism have been previously demonstrated in the brain of PD patients (98). It has been reported that Parkinson disease is characterized by chronic microglia activation. Microglia activation induces release of neurotoxic factors such as ROS, inflammatory cytokines including INF-γ which are potent inducers of KP. Activated KP in microglia upregulates production of neurotoxic KP metabolites such as 3-HK and QUIN (72). Moreover, increased KYNA/TRP ratio in CSF and serum together with increased level of 3-HK induces oxidative damage in dopamine neurons in the substantia nigra pars compacta (SNpc). These data suggest that reduced levels of KYNA are ineffective to block NMDA receptors and compensate neurotoxicity caused by 3-HK (99). Finally, excessive production of neurotoxic KP substances causes neuronal death and contributes to the progression of Parkinson disease. Similar to AD, our main result showed that blood levels of TRP were significantly decreased in PD patients compared to controls (SMD=-0.77, 95% CI=-1.24 to -0.30, p=0.001, I2 = 74.9%, k=4, n=352) (**Table 5**). KAT 1 to 4 are enzymes responsible for converting KYN to KYNA (100). An animal study reported that KAT 1 expression is reduced in the SNpc of MPTP-treated mice which subsequently leads to reduced level of KYNA in brain (101). Plasma samples of PD patients revealed considerably lower KAT 1 and KAT 2 activity (24) which could result in decreased KYNA concentrations. Overall, there were no significant differences between patients and controls in the blood levels of KYN, KYNA, AA, and 3-HK. This difference could be due to a small number of patients, or the effect of age, gender or other characteristics that could manipulate KP activation. In addition, plasma 3-HK was found to be strictly associated with both symptom severity and disease duration in PD patients (102). This highlights the effect of PD onset and disease stage on concentrations of KP compounds, though individual differences in activation of KP must be considered. In contrast to our findings, it has been claimed that PD patients show elevated serum levels of the KYN : TRP ratio, as well as elevated serum levels of AA, KYNA, and KYN, all compared to controls (103). In other study on PD rats and humans, plasma and CSF concentrations of TRP, KYN, and 3-HK were increased and induced oxidative stress (104–106). Nonetheless, we found no significant differences between patients and controls in the CSF levels of TRP, KYN, 5HIAA, and 3-HK. Lower levels of TRP found in PD patients indicates that KP intervention could relieve PD symptoms through neuroprotection (98). Accordingly, biomarker studies are necessary for early diagnosis of PD and they could provide pharmacological therapeutic opportunities.

### Huntington’s disease

Huntington disease is an autosomal dominant inherited disease caused by CAG tract expansion within exon 1 of the Huntington gene (HTT). Production of the number of CAG repeats beyond the threshold causes translated HTT proteins prone to dysfunction and neurotoxic aggregations (107). It has been demonstrated that abundant production of inflammatory cytokines such as interleukin 2 (IL-2) correlate with disease severity and increase of (K/T) ratio (52). Microglia play central role in CNS immune responses and their activation is associated with disease progression. The activation of neuroinflammatory response and microglia could further increase the activity of IDO and KP downstream (4). A recent study conducted on 40 premanifest HD patients, 40 manifest HD patients, and 20 HCs, denied the alteration of KP during HD pathogenesis (108). In contrast, we found that blood levels of TRP and KYN were significantly lower in HD patients than controls (SMD=-0.90, 95% CI=-1.71 to -0.10, p=0.028, I2 = 91.0%, k=5, n=369) (**Table 6**). Blood levels of 3- HANA were not significantly different between patients and controls. Previous studies demonstrated higher plasma KYN : TRP and lower KYNA : KYN ratios compared with controls (23). According to our findings, levels of KYNA and 3-HK in the frontal cortex were not significantly different between patients and controls. However, a postmortem brain study showed significantly higher 3-HK levels in the frontal and temporal cortex in HD brain samples in comparison with HCs (109). Inconsistencies around the association of KP and HD pathogenesis illuminates the necessity to further investigate possible associated variables such as age, age-onset of HD, related medical treatments, pattern of disease progression, and HD manifestations.

Overall, the current study reports the changes in levels of KP metabolites among patients living with PD, AD, and HD. There are various reports considering this relation but due to limitations and insufficient controlled studies (e.g., for age, disease manifestations and progression), it is not yet possible to reach a definitive conclusion.

## Limitations

We acknowledge that the current systematic review and meta-analysis has limitations. Most included studies did not describe the stage of AD, HD, or PD; therefore, we were not able to examine the stage-associated alterations. Several studies did not precisely determine therapies which could affect KP activation. Importantly, the lack of postmortem studies and small sample sizes involving CSF studies limit the ability to draw general conclusions on KP metabolite concentrations in the brains affected by the three considered diseases. Moreover, the effects of age, disease duration, and inflammatory conditions such as viral, fungal, parasite or bacterial infections leading to sepsis status of the body, inflammatory systemic diseases including rheumatoid arthritis, autoimmune encephalitis, stress and other psychiatric conditions, and pregnancy (110–113) were not considered by the included studies. Variability in plasma levels of tryptophan metabolites could result from the time of the day or fasting status of participants. However, just some studies reported the fasting status of participants available data reported that participants entered study in fasting state. For better elaboration it is required to have fasting status of all entered participants and compare the effect of different fasting status on different KP metabolites.

## Conclusion

Finally, as shown in the results, the blood level of TRP was lower in the AD, PD, and HD patients in comparison to the healthy controls. Also, the levels of 3-HK in the CSF of AD patients and KYN levels in the blood of HD patients were lower than the respective controls. These outcomes suggest that the KP may be altered during the neurodegenerative processes taking place in AD, PD, and HD patients, and the concentrations of resulting metabolites can be affected. Furthermore, it is proposed that the levels of these metabolites depend on the stage of the neurodegenerative disease, and it has been suggested that more studies assessing the KP at different stages of AD, PD, and HD should be designed.

In conclusion, although some alterations have been observed, this meta-analysis indicate that the measure of KP metabolism does not find a precise utilization in the definition of neurodegenerative disorders pathogenesis. However, more studies are required to conclusively establish alteration of KP metabolites in neurodegenerative diseases. Understanding the exact role of KP in these neurodegenerative diseases could identify strategies for preventing the neurodegeneration or slowing down its progress, as well as conducting pre-symptomatic diagnosis of AD, PD, and HD.

## Data availability statement

The original contributions presented in the study are included in the article/**Supplementary Material**. Further inquiries can be directed to the corresponding authors.

## Author contributions

MF, KV, and SY contributed to the conception and design of the study. MR and FSa contributed to the supervision of the manuscript. AT and RH organized the database. AK and AM edited the paper scientifically. MF and SY analyzed the data. All authors wrote the first draft of the manuscript, wrote sections of the manuscript, contributed to manuscript revision, read, and approved the submitted version.

## Funding

This study is related to the project NO. (1399/61325) from Student Research Committee, Shahid Beheshti University of Medical Sciences, Tehran, Iran. We also appreciate the “Student Research Committee” and “Research & Technology Chancellor” in Shahid Beheshti University of Medical Sciences as well as the Jack Brown and Family Alzheimer’s Disease Research Foundation for their financial support of this study.

## Conflict of interest

The authors declare that the research was conducted in the absence of any commercial or financial relationships that could be construed as a potential conflict of interest.

## Publisher’s note

All claims expressed in this article are solely those of the authors and do not necessarily represent those of their affiliated organizations, or those of the publisher, the editors and the reviewers. Any product that may be evaluated in this article, or claim that may be made by its manufacturer, is not guaranteed or endorsed by the publisher.
