# Supplementary material for: Dynamic changes in metabolites of the kynurenine pathway in Alzheimer’s disease, Parkinson’s disease, and Huntington’s disease: A systematic Review and meta-analysis
Source: Front Immunol. 2022 Oct 3;13:997240. doi: 10.3389/fimmu.2022.997240 (PMC9574226; doi:10.3389/fimmu.2022.997240)
Supplement: Supplementary file 1 [file DataSheet_1.docx]

| Author (year) | Selection | | | | Comparability | Outcome | |
| --- | --- | --- | --- | --- | --- | --- | --- |
| Gonzalez-sanchez (2020)[1] | * |  |  | ** | ** | ** | * |
| Iwaoka (2020)[2] | * |  | * | ** |  | ** | * |
| Sorgdrager (2019)[3] | * |  | * | ** | ** | ** | * |
| Chang (2018)[4] | * |  | * | ** | ** | ** | * |
| Havelund (2017)[5] | * |  | * | ** |  | ** | * |
| Oxenkrug (2017)[6] | * |  | * | ** |  | ** | * |
| Giil (2017)[7] | * |  | * | ** |  | ** | * |
| Forrest (2010)[8] | * |  | * | ** |  | ** | * |
| Kaddurah-Daouk (2010)[9] | * |  | * | ** |  | ** | * |
| Gulaj (2009)[10] | * |  | * | ** |  | ** | * |
| Hartai (2007)[11] | * |  | * | ** |  | ** |  |
| Christofides (2006)[12] | * |  | * | ** |  | ** | * |
| Hartai (2005)[13] | * |  | * | ** | ** | ** |  |
| Guidetti (2004)[14] | * |  |  | ** |  | ** |  |
| Widner (2000)[15] | * |  |  | ** |  | ** | * |
| Bonaccorso (1998)[16] | * |  |  | ** | ** | ** |  |
| Fekkes (1998)[17] | * |  |  | ** |  | ** | * |
| Baran (1998)[18] | * |  | * | ** |  | ** | * |
| Molina (1997)[19] | * |  | * | ** |  | ** |  |
| Jauch (1995)[20] | * |  | * | ** |  | ** |  |
| Ruiz (1995) [21] | * |  | * | ** |  | ** |  |
| Tohgi H. (1995)[22] | * |  | * | ** |  | ** |  |
| Tohgi H. (1993)[23] | * |  | * | ** |  | ** |  |
| Tohgi (1993)[24] | * |  | * | ** |  | ** |  |
| Beal (1992)[25] | * |  | * | ** |  | ** | * |
| Pearson (1992)[26] | * |  | * | ** | ** | ** | * |
| Kay (1986)[27] | * |  |  | ** |  | ** | * |
| Belendiuk (1980)[28] | * |  | * | ** |  | ** |  |

1. González-Sánchez, M., et al., *Kynurenic acid levels are increased in the CSF of Alzheimer’s disease patients.* Biomolecules, 2020. **10**(4): p. 571.

2. Iwaoka, K., et al., *Impaired metabolism of kynurenine and its metabolites in CSF of parkinson’s disease.* Neuroscience Letters, 2020. **714**: p. 134576.

3. Sorgdrager, F.J., et al., *Age‐and disease‐specific changes of the kynurenine pathway in Parkinson’s and Alzheimer’s disease.* Journal of Neurochemistry, 2019. **151**(5): p. 656-668.

4. Chang, K.-H., et al., *Alternations of metabolic profile and kynurenine metabolism in the plasma of Parkinson’s disease.* Molecular neurobiology, 2018. **55**(8): p. 6319-6328.

5. Havelund, J.F., et al., *Changes in kynurenine pathway metabolism in Parkinson patients with L‐DOPA‐induced dyskinesia.* Journal of neurochemistry, 2017. **142**(5): p. 756-766.

6. Oxenkrug, G., et al., *Peripheral tryptophan-kynurenine metabolism associated with metabolic syndrome is different in Parkinson’s and Alzheimer’s diseases.* Endocrinology, diabetes and metabolism journal, 2017. **1**(4).

7. Giil, L.M., et al., *Kynurenine pathway metabolites in Alzheimer’s disease.* Journal of Alzheimer's Disease, 2017. **60**(2): p. 495-504.

8. Forrest, C.M., et al., *Blood levels of kynurenines, interleukin‐23 and soluble human leucocyte antigen‐G at different stages of Huntington’s disease.* Journal of neurochemistry, 2010. **112**(1): p. 112-122.

9. Kaddurah-Daouk, R., et al., *Alterations in metabolic pathways and networks in Alzheimer’s disease.* Translational psychiatry, 2013. **3**(4): p. e244-e244.

10. Gulaj, E., et al., *Kynurenine and its metabolites in Alzheimer's disease patients.* Advances in Medical Sciences, 2010. **55**(2): p. 204-211.

11. Hartai, Z., et al., *Decreased serum and red blood cell kynurenic acid levels in Alzheimer's disease.* Neurochemistry international, 2007. **50**(2): p. 308-313.

12. Christofides, J., et al., *Blood 5‐hydroxytryptamine, 5‐hydroxyindoleacetic acid and melatonin levels in patients with either Huntington's disease or chronic brain injury.* Journal of neurochemistry, 2006. **97**(4): p. 1078-1088.

13. Hartai, Z., et al., *Kynurenine metabolism in plasma and in red blood cells in Parkinson's disease.* Journal of the neurological sciences, 2005. **239**(1): p. 31-35.

14. Guidetti, P., et al., *Neostriatal and cortical quinolinate levels are increased in early grade Huntington's disease.* Neurobiology of disease, 2004. **17**(3): p. 455-461.

15. Widner, B., et al., *Tryptophan degradation and immune activation in Alzheimer's disease.* Journal of neural transmission, 2000. **107**(3): p. 343-353.

16. Bonaccorso, S., et al., *Serotonin-immune interactions in elderly volunteers and in patients with Alzheimer’s disease (DAT): lower plasma tryptophan availability to the brain in the elderly and increased serum interleukin-6 in DAT.* Aging Clinical and Experimental Research, 1998. **10**(4): p. 316-323.

17. Fekkes, D., et al., *Abnormal amino acid metabolism in patients with early stage Alzheimer dementia.* Journal of Neural Transmission, 1998. **105**(2): p. 287-294.

18. Baran, H., K. Jellinger, and L. Deecke, *Kynurenine metabolism in Alzheimer's disease.* Journal of neural transmission, 1999. **106**(2): p. 165-181.

19. Molina, J.A., et al., *Decreased cerebrospinal fluid levels of neutral and basic amino acids in patients with Parkinson's disease.* Journal of the neurological sciences, 1997. **150**(2): p. 123-127.

20. Jauch, D., et al., *Dysfunction of brain kynurenic acid metabolism in Huntington's disease: focus on kynurenine aminotransferases.* Journal of the neurological sciences, 1995. **130**(1): p. 39-47.

21. Ruiz, P.G., et al., *Cerebrospinal fluid homovanillic acid is reduced in untreated Huntington's disease.* Clin. Neuropharmacol., 1995. **18**(1): p. 58-63.

22. Tohgi, H., et al., *Alterations in the concentration of serotonergic and dopaminergic substances in the cerebrospinal fluid of patients with Parkinson's disease, and their changes after L-dopa administration.* Neuroscience letters, 1993. **159**(1-2): p. 135-138.

23. Tohgi, H., et al., *Concentrations of serotonin and its related substances in the cerebrospinal fluid in patients with Alzheimer type dementia.* Neuroscience letters, 1992. **141**(1): p. 9-12.

24. Tohgi, H., et al., *Concentrations of serotonin and its related substances in the cerebrospinal fluid of parkinsonian patients and their relations to the severity of symptoms.* Neuroscience letters, 1993. **150**(1): p. 71-74.

25. Beal, M.F., et al., *Kynurenic acid concentrations are reduced in Huntington's disease cerebral cortex.* Journal of the neurological sciences, 1992. **108**(1): p. 80-87.

26. Pearson, S. and G. Reynolds, *Increased brain concentrations of a neurotoxin, 3-hydroxykynurenine, in Huntington's disease.* Neuroscience letters, 1992. **144**(1-2): p. 199-201.

27. Kay, A.D., et al., *Cerebrospinal fluid biopterin is decreased in Alzheimer's disease.* Archives of neurology, 1986. **43**(10): p. 996-999.

28. Belendiuk, K., G.W. Belendiuk, and D.X. Freedman, *Blood monoamine metabolism in Huntington's disease.* Archives of general psychiatry, 1980. **37**(3): p. 325-332.


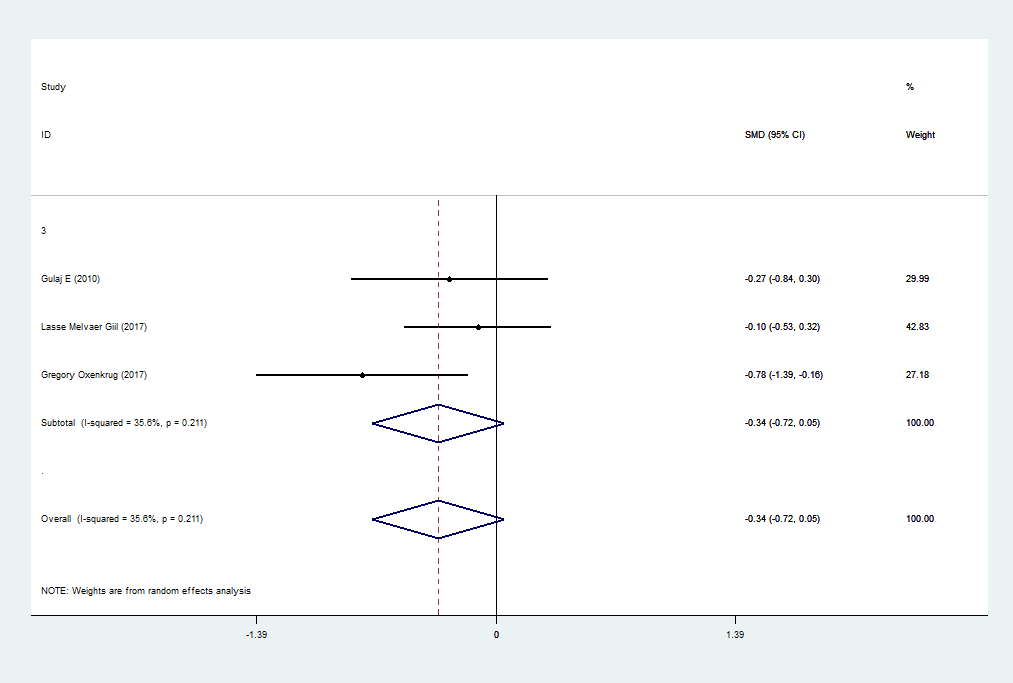


**Figure 3d. Forest plot of the levels of ANT in AD patients.** In this plot, the squares are applied to show the mean effect estimate of each paper along with their 95% CI. The size of each square is considered proportional to the weight of the parameter in the meta-analysis, and is also demonstrated in a separate column. (1: temporal cortex/hippocampi; 2:CSF; 3:blood(serum/plasma); 4:serum; 5:plasma)


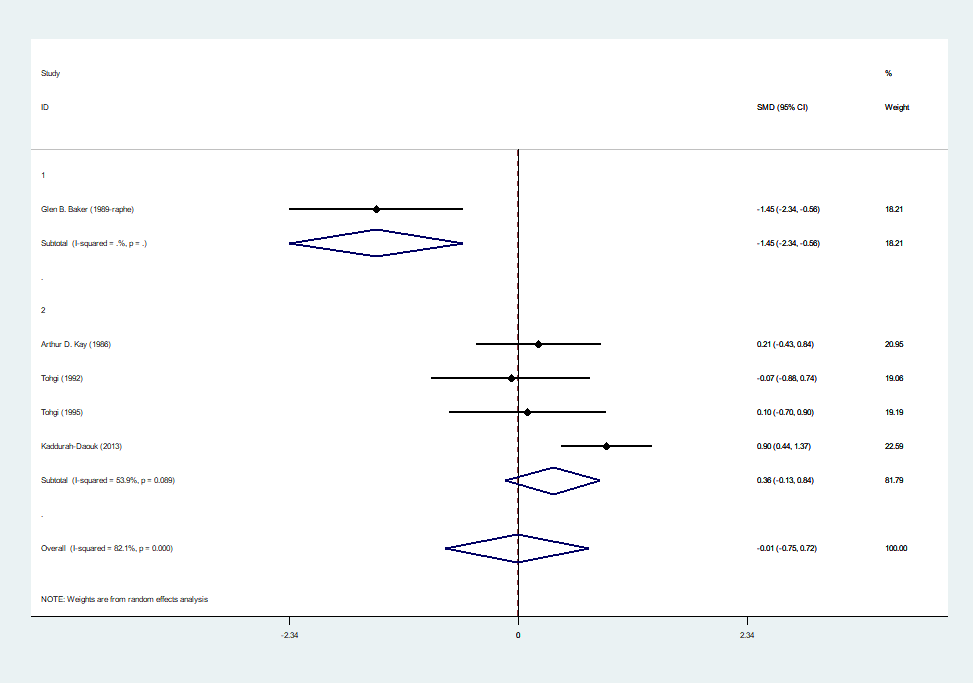


**Figure 3e. Forest plot of the levels of 5-HIAA in AD patients.** In this plot, the squares are applied to show the mean effect estimate of each paper along with their 95% CI. The size of each square is considered proportional to the weight of the parameter in the meta-analysis, and is also demonstrated in a separate column. (1: temporal cortex/hippocampi; 2:CSF; 3:blood(serum/plasma); 4:serum; 5:plasma)


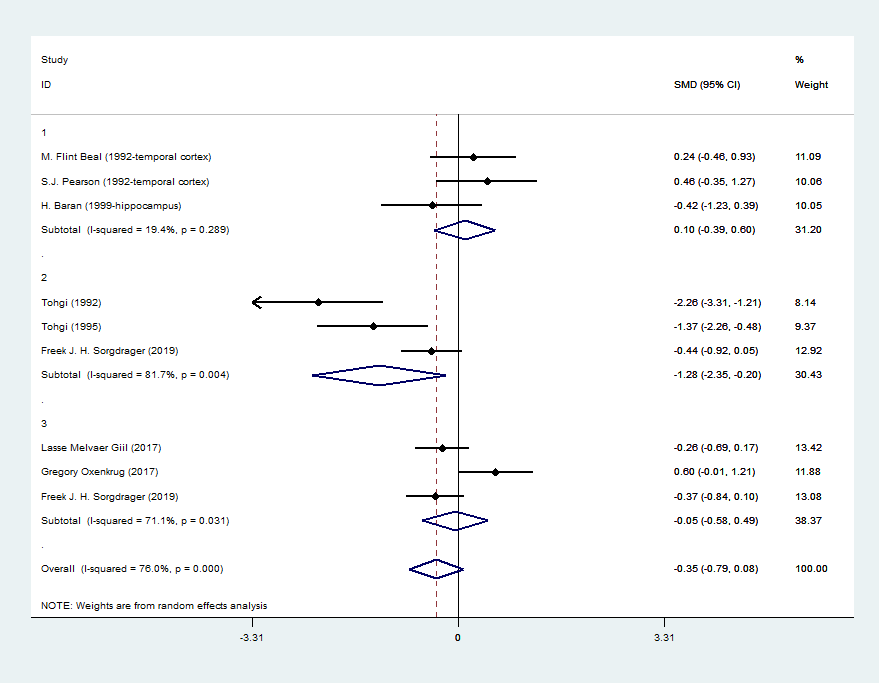


**Figure 3f. Forest plot of the levels of 3-HK in AD patients.** In this plot, the squares are applied to show the mean effect estimate of each paper along with their 95% CI. The size of each square is considered proportional to the weight of the parameter in the meta-analysis, and is also demonstrated in a separate column. (1: temporal cortex/hippocampi; 2:CSF; 3:blood(serum/plasma); 4:serum; 5:plasma)


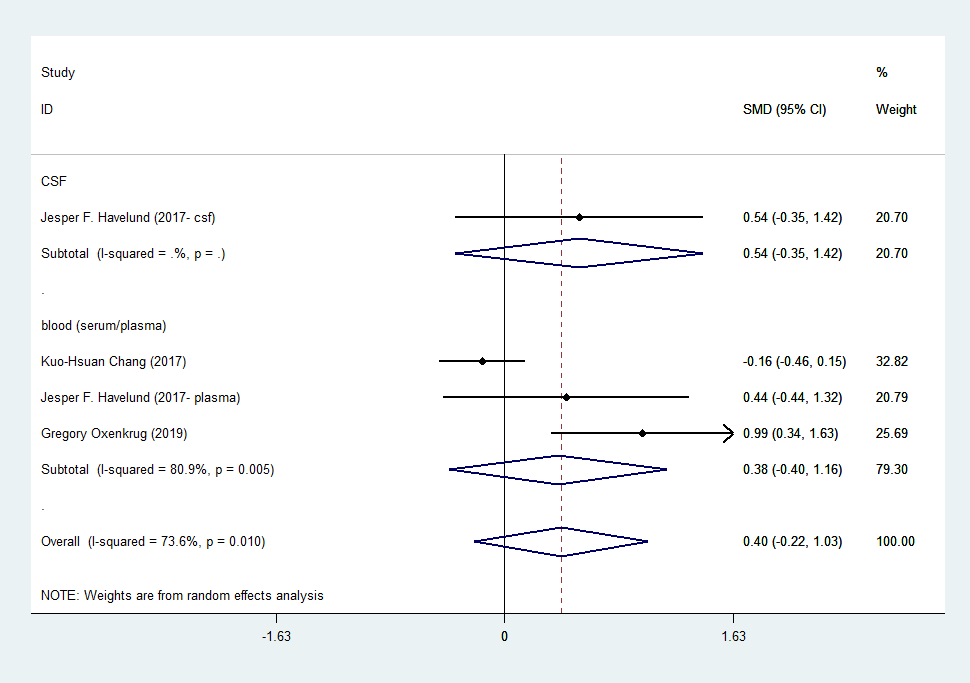


**Figure 4d. Forest plot of the levels of ANT in PD patients.** In this plot, the squares are applied to show the mean effect estimate of each paper along with their 95% CI. The size of each square is considered proportional to the weight of the parameter in the meta-analysis, and is also demonstrated in a separate column.


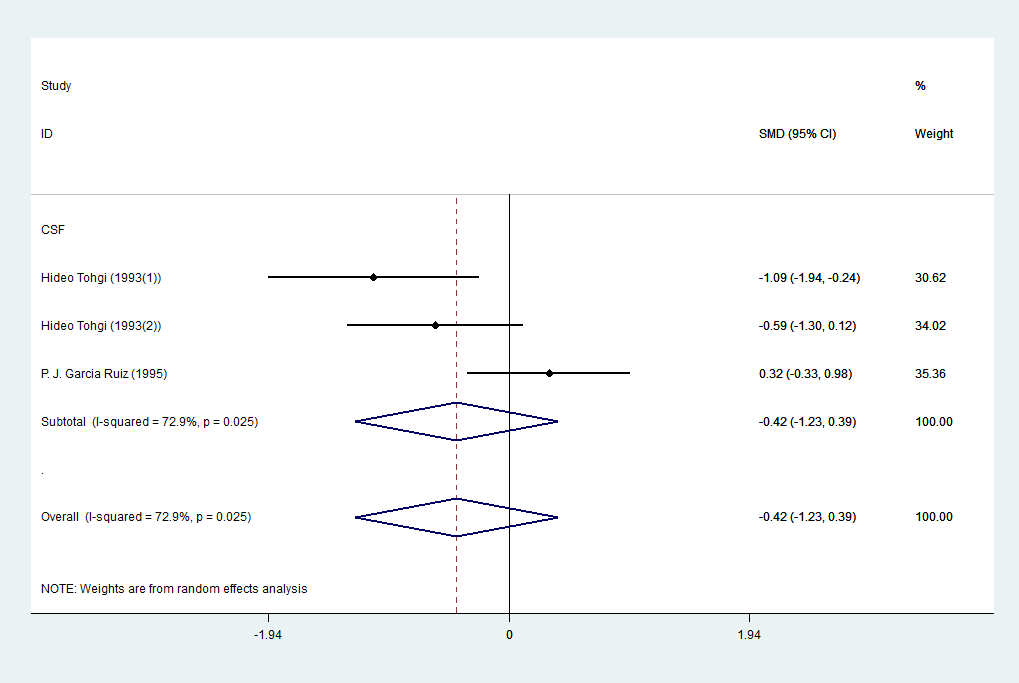


**Figure 4e. Forest plot of the levels of 5-HIAA in PD patients.** In this plot, the squares are applied to show the mean effect estimate of each paper along with their 95% CI. The size of each square is considered proportional to the weight of the parameter in the meta-analysis, and is also demonstrated in a separate column.


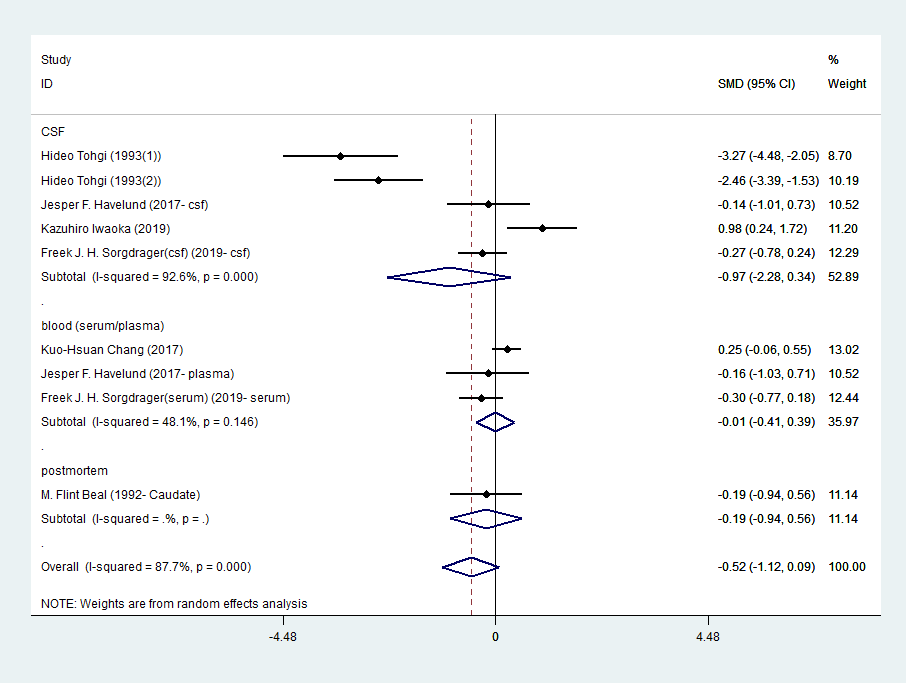


**Figure 4f. Forest plot of the levels of 3-HK in PD patients.** In this plot, the squares are applied to show the mean effect estimate of each paper along with their 95% CI. The size of each square is considered proportional to the weight of the parameter in the meta-analysis, and is also demonstrated in a separate column.


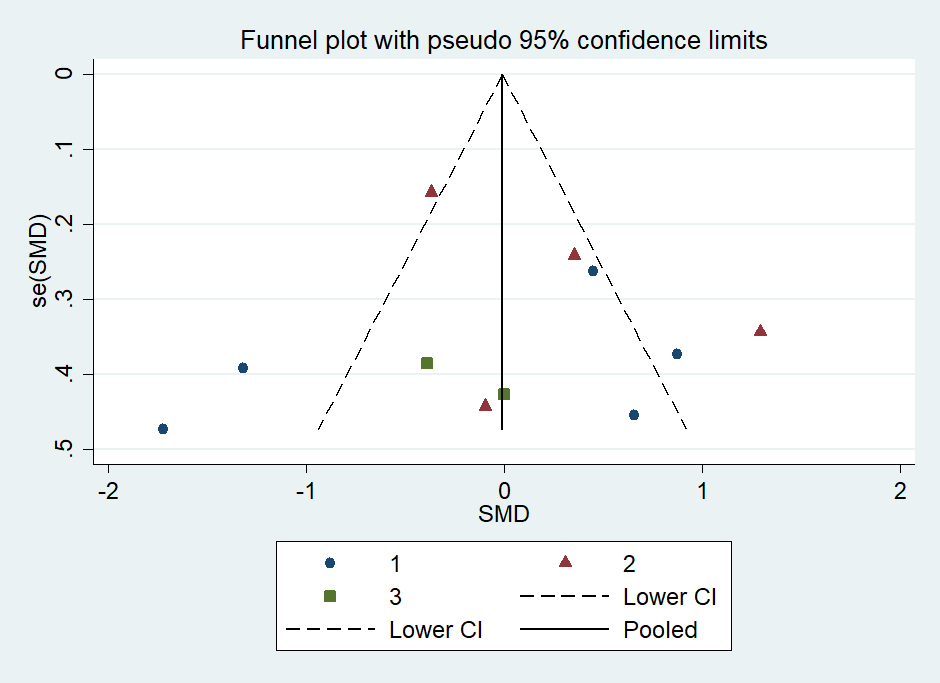


**Fig.4g. Funnel plot for publication bias with pseudo 95% confidence limits for PD based on TRP**


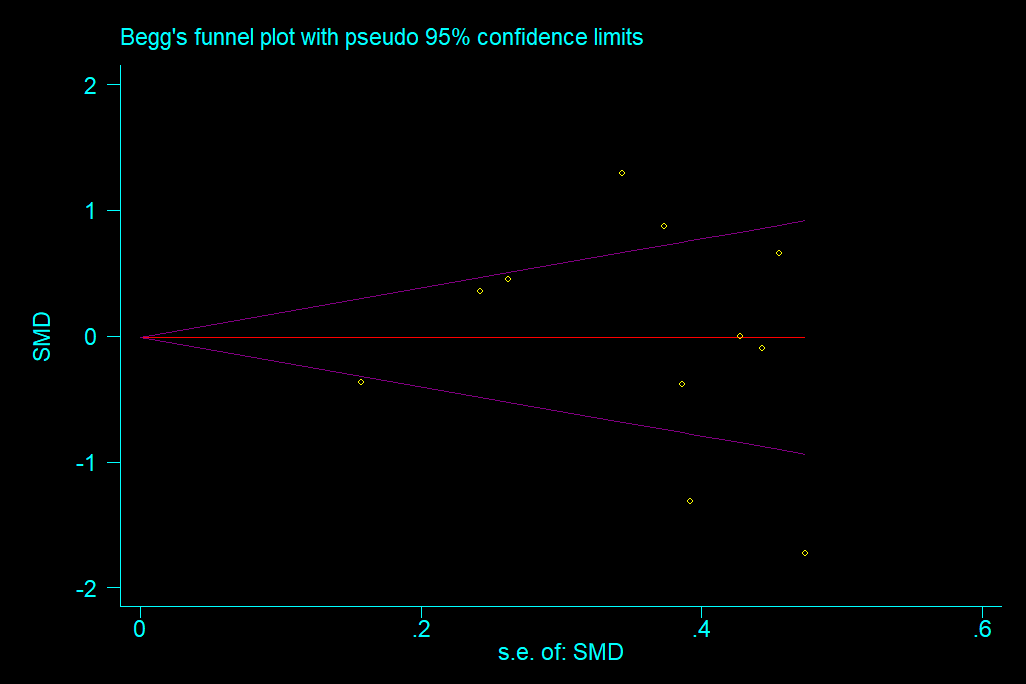


**Fig.4h. Begg’s funnel plot for publication bias with pseudo 95% confidence limits for PD**


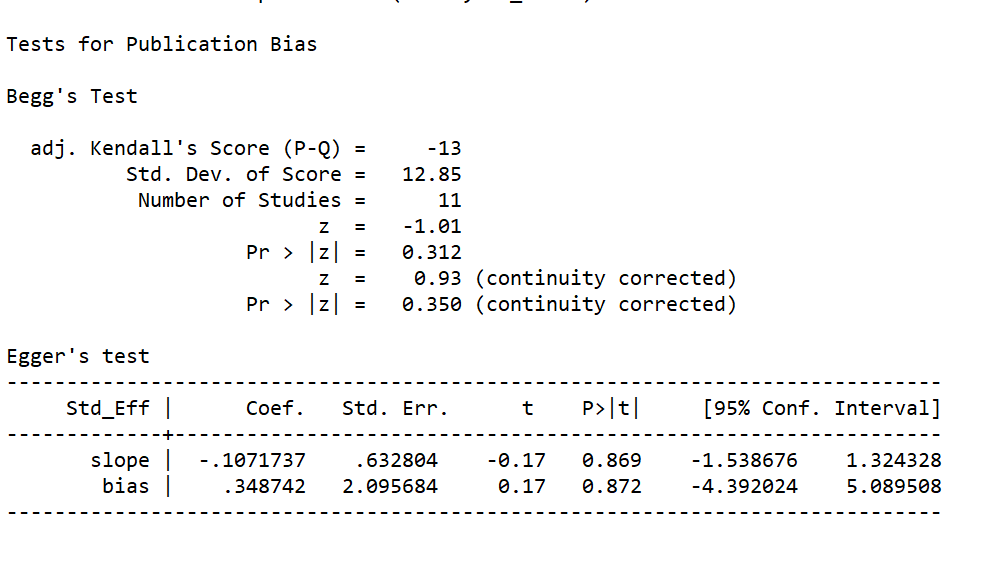


**Fig.4i. Begg and Egger’s test for publication bias with pseudo 95% confidence limits for PD**


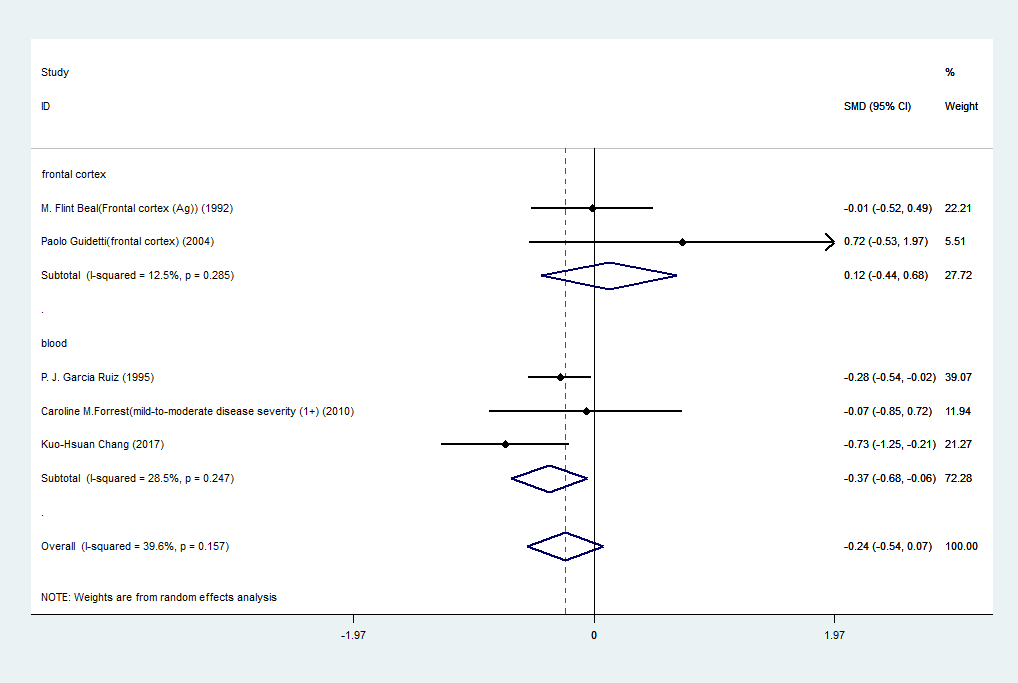


**Figure 5b. Forest plot of the levels of KYN in HD patients.** In this plot, the squares are applied to show the mean effect estimate of each paper along with their 95% CI. The size of each square is considered proportional to the weight of the parameter in the meta-analysis, and is also demonstrated in a separate column.


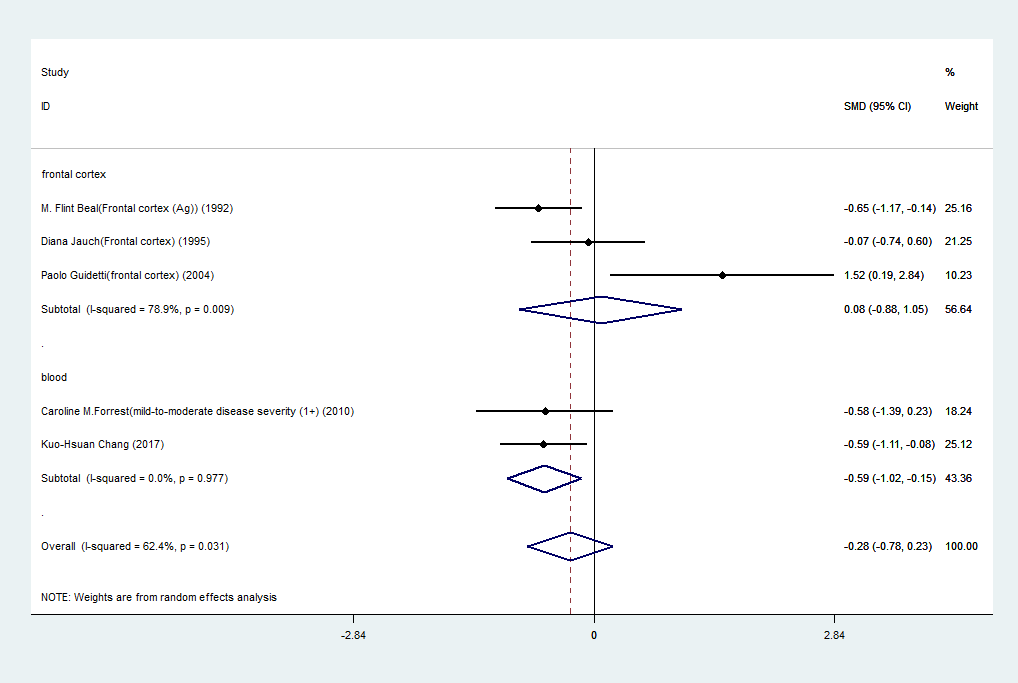


**Figure 5c. Forest plot of the levels of KYNA in HD patients.** In this plot, the squares are applied to show the mean effect estimate of each paper along with their 95% CI. The size of each square is considered proportional to the weight of the parameter in the meta-analysis, and is also demonstrated in a separate column.


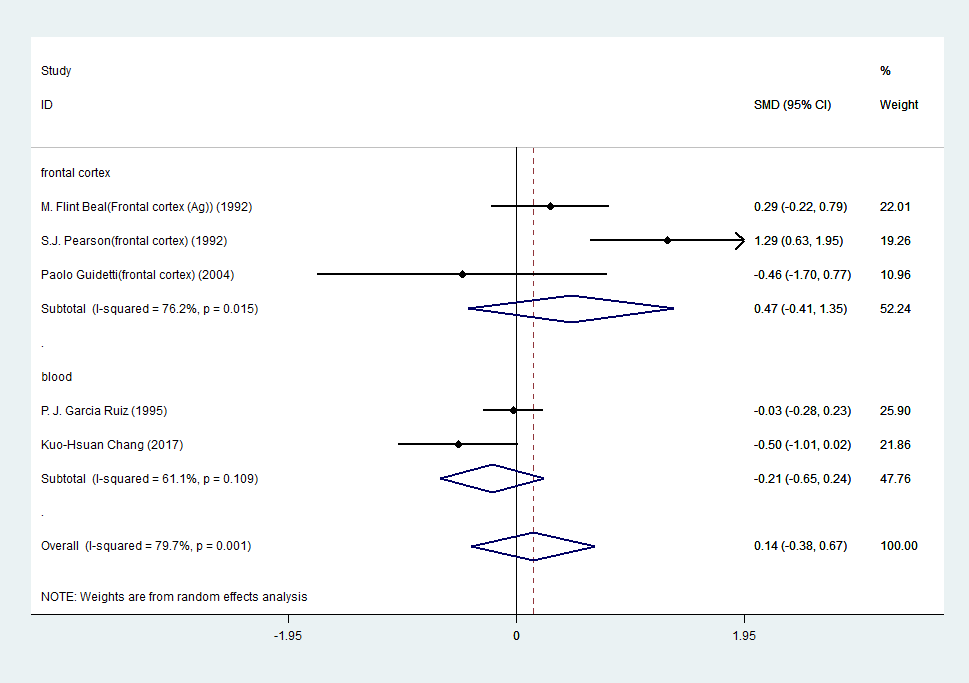


**Figure 5d. Forest plot of the levels of 3-HK in HD patients.** In this plot, the squares are applied to show the mean effect estimate of each paper along with their 95% CI. The size of each square is considered proportional to the weight of the parameter in the meta-analysis, and is also demonstrated in a separate column.


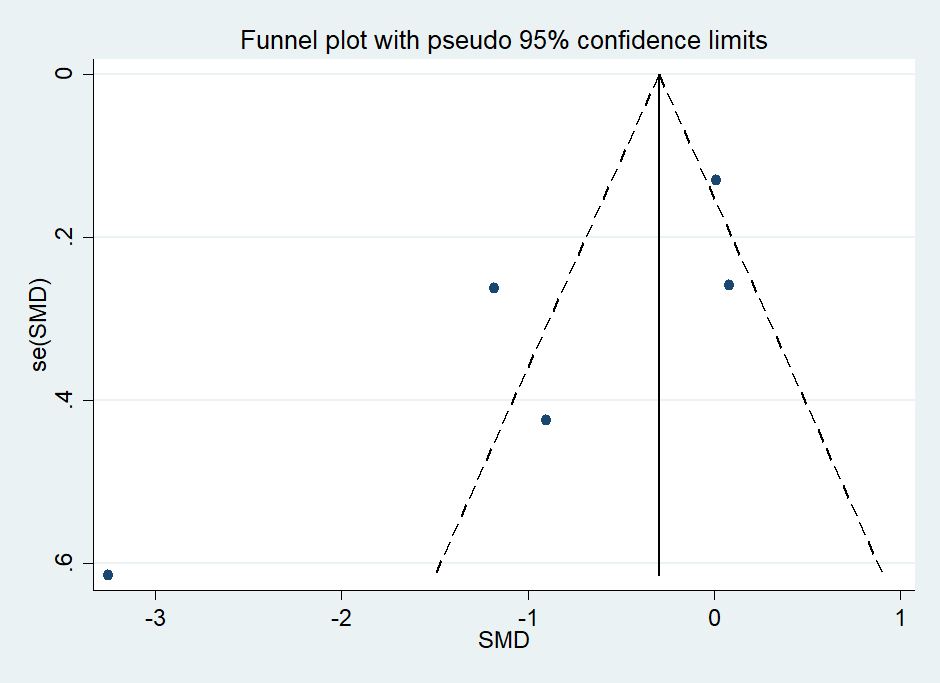


**Fig.5e. Funnel plot for publication bias with pseudo 95% confidence limits for HD based on TRP**


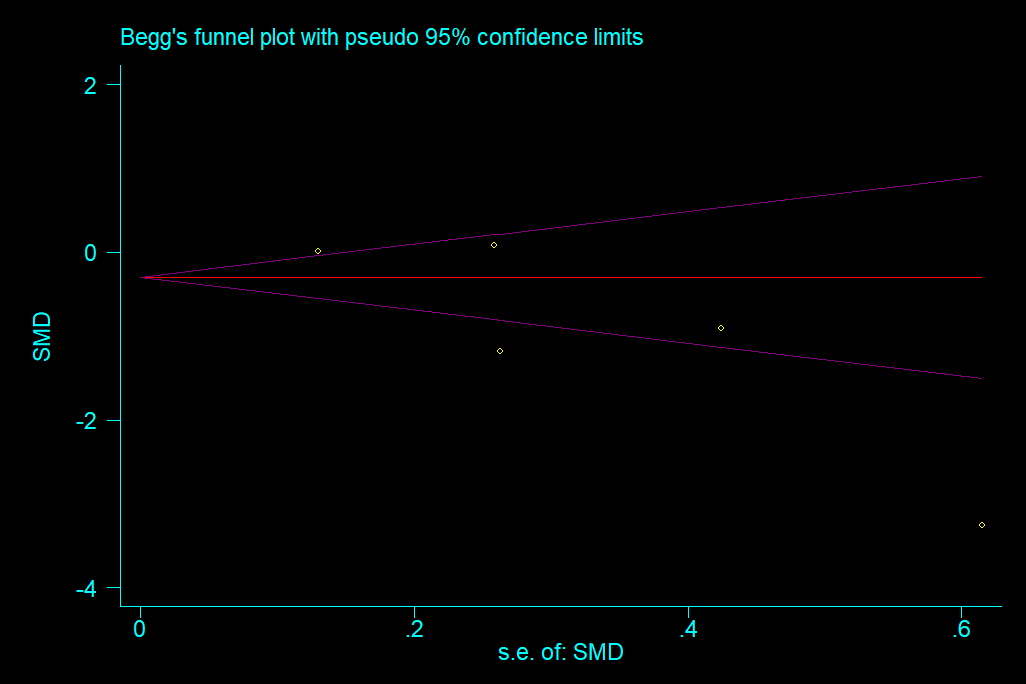


**Fig.5f. Begg’s funnel plot for publication bias with pseudo 95% confidence limits for HD**


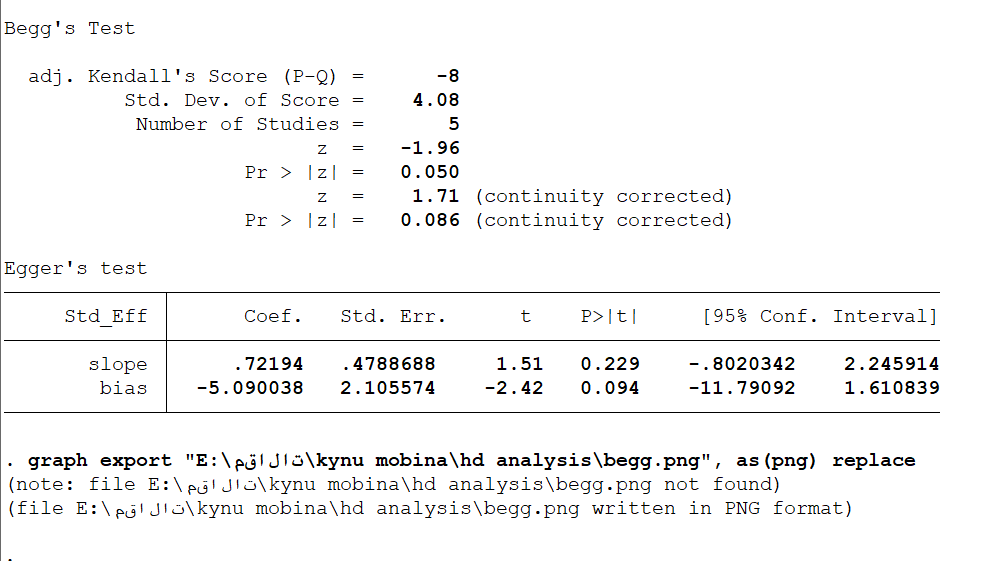


**Fig.5g. Begg and Egger’s test for publication bias with pseudo 95% confidence limits for HD**
